# Transcranial magnetic stimulation maps the neurophysiology of chronic noncancer pain: A scoping review

**DOI:** 10.1097/MD.0000000000031774

**Published:** 2022-11-18

**Authors:** Nicholas Jacob Snow, Megan Christine Kirkland, Matthew Bruce Downer, Hannah Margaret Murphy, Michelle Ploughman

**Affiliations:** a Recovery and Performance Laboratory, Faculty of Medicine, Memorial University of Newfoundland & Labrador, St. John’s, NL, Canada.

**Keywords:** biomarker, chronic pain, scoping review, transcranial magnetic stimulation

## Abstract

Chronic noncancer pain is a global public health challenge. It is imperative to identify biological markers (“biomarkers”) to understand the mechanisms underlying chronic pain and to monitor pain over time and after interventions. Transcranial magnetic stimulation (TMS) is a promising method for this purpose.

**Objectives:**

To examine differences in TMS-based outcomes between persons with chronic pain and healthy controls (HCs) and/or before versus after pain-modulating interventions and relationships between pain measures and TMS outcomes; To summarize the neurophysiological mechanisms underlying chronic pain as identified by TMS.

**Methods:**

We searched the PubMed database for literature from January 1, 1985, to June 9, 2020, with the keywords “pain” and “transcranial magnetic stimulation.” Eligible items included original studies of adult human participants with pain lasting for ≥ 6 months. We completed a narrative synthesis of the study findings stratified by chronic pain etiology (primary pain, neuropathic pain, and secondary musculoskeletal pain).

**Results:**

The search yielded 1265 records. The final 12 articles included 244 patients with chronic pain (192 females, aged 35‐65 years) and 169 HCs (89 females, aged 28‐59 years). Abnormalities in TMS outcomes that reflect GABAergic and glutamatergic activities were associated with many of the disorders studied and were distinct for each pain etiology. Chronic primary pain is characterized by reduced intracortical inhibition and corticospinal excitability, chronic neuropathic pain shows evidence of increased excitation and disinhibition, and chronic secondary musculoskeletal pain involves low corticospinal excitability.

**Discussion:**

TMS could be a useful tool for delineating the neurophysiological underpinnings of chronic pain syndromes.

## 1. Introduction

Chronic non-cancer pain encompasses a collection of syndromes that are associated with poor functional status and quality of life. Worldwide, the prevalence of chronic noncancer pain in adults is 10% to 25%,^[1–4]^ while its incidence is estimated at 10% per year.^[4]^ Furthermore, A study of primary care practices in Finland found that up to 1-half of physician visits were related to chronic pain.^[5]^ In the United States, the annual combined economic costs of chronic pain exceed $560 billion.^[3]^ Due of its high prevalence and impact on physical disability, psychosocial dysfunction, and addiction, chronic pain is a global public health crisis.^[3,4]^

Chronic pain is defined as lasting longer than 3 to 6 months in duration, or beyond the time required for normal tissue healing after acute injury.^[6,7]^ Depending on its etiology, chronic pain may persist in the absence of identifiable evidence of tissue damage.^[8]^ In contrast to nociception, which involves the objective identification of noxious stimulation of pain receptors and afferent nerves, pain is a more subjective phenomenon that may or may not result from direct sensory stimulation.^[6,9]^ Typically, the longer the duration of chronic pain, the greater the affective consequences, such as anxiety and depression, and the more significant and pervasive the associated neural changes.^[10–12]^ Due to the high prevalence of chronic pain and its negative long-term health outcomes, identifying effective treatments is of paramount priority.^[3]^

A significant challenge in treating chronic non-cancer pain is the complexity of the pathophysiology and variety of etiologies. The international statistical classification of diseases and related health problems, 11^th^ edition (ICD-11) enables a relatively streamlined identification of chronic noncancer pain syndromes based on the underlying mechanism or etiology of diagnosis.^[7,13,14]^ ICD-11 classifications include, for example, neuropathic (i.e., caused by a lesion or disease involving the somatosensory nervous system),^[14]^ musculoskeletal (i.e., involving a disease process directly affecting bone, joint, muscle, or other soft tissue),^[15]^ and primary pain (i.e., from an unknown source).^[7]^ Nevertheless, despite a move towards a more standardized classification of chronic pain, the large degree of heterogeneity in chronic pain presentations is a key challenge in providing treatment.

Despite the etiological complexity of chronic pain, experimental studies suggest that aberrant glutamatergic and γ-aminobutyric acid (GABA)-ergic signaling in the central nervous system (CNS) may be a common factor in chronic pain and a putative target for study and intervention.^[10,16–24]^ Many first-line agents for the pharmacotherapy of chronic pain mimic the structure of GABA.^[20,21,25–28]^ Nevertheless, in humans, little direct evidence exists to help guide chronic pain manipulation because of the inability to directly visualize the CNS, as well as a paucity of biological markers (“biomarkers”) that reflect chronic pain pathophysiology.^[20]^

Biomarkers can be used to aid diagnosis, clarify disease pathophysiology, classify the extent of a disease, indicate disease prognosis, and predict or monitor the disease over time and in response to interventions.^[29]^ One proposed biomarker for assessing CNS pathophysiology in chronic pain is transcranial magnetic stimulation (TMS).^[30]^ TMS uses electromagnetic induction to noninvasively generate an electrical current in the brain.^[31–33]^ TMS involves a high-current pulse generator that discharges an electrical current of several thousand amperes through an insulated metal stimulating coil for a period of < 1ms, generating a brief and focal magnetic field of approximately 1 to 2 Tesla.^[32–34]^ When the coil is placed on an individual’s scalp overlying the primary motor cortex (M1), the magnetic field can induce an intracortical electric current sufficient to depolarize superficial corticospinal neurons and activate a target muscle leading to a measurable electromyographic response.^[31–34]^ The neurotransmitters involved in various TMS-evoked measurements have been well characterized,^[24,35–37]^ thus making TMS a putative biomarker for the study of chronic pain.

This work endeavored to synthesize evidence related to the use of TMS as a pathophysiological biomarker of chronic non-cancer pain. We used the ICD-11 classification to stratify the studies based on chronic pain etiology.

## 2. Materials and methods

We registered our scoping review protocol with the Center for Open Science Database (ID: VP72N). We consulted the Preferred Reporting Items for Systematic Review and Meta-Analysis Protocols and Scoping Reviews checklists,^[38–41]^ published guidelines for scoping reviews,^[42,43]^ and Cochrane Collaboration’s Synthesis without Meta-Analysis recommendations.^[44,45]^ We did not complete an institutional ethics review board application because no participants were directly involved in the study.

### 2.1. Identifying the research question

This scoping review aimed to explore the role of TMS as a pathophysiological biomarker for characterization and monitoring of chronic pain syndromes. Our objectives were to identify evidence on (i) differences in TMS-based outcomes between persons with chronic pain and healthy controls (HCs) and/or before versus after pain-modulating interventions, (ii) relationships between quantitative pain measures and TMS outcomes, and summarize neurophysiological mechanisms underlying chronic pain, as identified by TMS.

### 2.2. Search strategy

We searched the PubMed database for relevant literature from January 1, 1985 (the inaugural year of TMS publication^[34]^) to June 9, 2020. The following keywords were used: (“pain”[MeSH] OR “pain”[tiab]) AND (“transcranial magnetic stimulation”[MeSH] OR “transcranial magnetic stimulation”[tiab] OR “TMS”[tiab]). Furthermore, any relevant review articles were flagged, and their reference lists were scanned to identify additional relevant records. We considered only peer-reviewed, full-text research manuscripts published in English.

### 2.3. Study selection

All records were imported into Covidence software (Veritas Health Innovation, Melbourne, Victoria, Australia).^[46]^ The study selection involved initial title and abstract screening, followed by full-text review. If full-text articles could not be obtained, the authors of the manuscript were contacted. Records were weighed against the eligibility criteria (Table 1) by 2 independent raters in duplicate (MCK and NJS).^[47]^ Discrepancies were resolved by consensus.

**Table 1:** Study eligibility criteria, based on PICO (Population, Intervention, Control/Comparison, Outcome) criteria.^[47]^

Component	Inclusion criteria	Exclusion criteria
Population	● Human participants. ● Chronic non-cancer pain syndromes operationalized as pain lasting > 6 months and not associated with an acute illness or injury.	● Experimentally induced pain. ● Acute pain.
Intervention	● Any intervention. ● Observational studies with no intervention.	─
Control/ Comparison	● Studies using a control condition (for interventional studies) or healthy control group (for observational studies).	● Studies that do not employ a control condition or group; that utilize a crossover design without a true control group or condition; or that utilize an intervention in both the patient and healthy control groups, without a true control group or condition.
Outcomes	● Measure to quantify pain: reported as primary or secondary outcome measures. ● Any single- or paired-pulse TMS-evoked EMG (upper and lower extremity skeletal muscles directly innervated by spinal nerves; surface electrodes) or EEG (scalp electrodes) potentials.	● No quantification of pain. ● rTMS interventions with no TMS-based outcomes measures. ● TMS-evoked EMG, measures using indwelling electrodes or high-density surface arrays. ● TMS-evoked EEG measures involving power band oscillations. ● EMG measurements from non-extremity muscles (e.g., facial, pelvic, truncal, or anorectal skeletal or smooth muscles) or muscles innervated by cranial nerves (e.g., facial muscles, trapezius).
Study Design	● Quantitative, original, research studies. ● Full-text manuscripts from peer-reviewed academic journals. ● Interventional (e.g., randomized, or non-randomized controlled trials) or observational (e.g., cross-sectional) studies.	● Reviews papers, perspective papers, commentaries, and letters to the editor will not be included. ● Case studies and case series will not be included. ● Records for which a full-text article could not be obtained. ● Within-subjects cross-sectional designs without a separate designated control group or condition will not be included.
Language	● English	─

EEG = electroencephalogram, EMG = electromyogram, TMS = transcranial magnetic stimulation, rTMS = repetitive transcranial magnetic stimulation.

### 2.4. Charting the data

Data from the full-text articles were transcribed into a standardized data extraction form (Microsoft Excel, Redmond, WA). Data of interest included participants (e.g., chronic pain syndrome, demographics, comorbidities, baseline treatments) and study characteristics (e.g., study design, intervention description), TMS methods and outcomes, quantitative pain measures, secondary clinical outcomes (e.g., depression, anxiety, quality of life), relationships between TMS and clinical outcomes, and the effects of interventions on TMS and clinical outcomes.

Data were extracted by 4 raters (MCK, NJS, MBD, and HMM). To establish our data charting method, we performed a charting and group consultation exercise.^[43,48]^ Raters independently charted 5 randomly selected studies (random number generator) and met to determine whether their approach was consistent with the research aims and reached consensus about the data collection process.^[43,48]^ Articles were randomly assigned to each rater using an online randomization tool (http://www.randomization.com).^[49]^ Extracted data were reviewed by 2 raters (MBD and NJS), who resolved discrepancies by consensus.

### 2.5. Collating, summarizing, and reporting results

We conducted a qualitative narrative synthesis, focusing on the objectives listed above. We summarized the key concepts related to TMS as a pathophysiological biomarker for chronic pain characterization and monitoring. We performed a general summary of the study findings with narrative subgroup analyses,^[50,51]^ using the ICD-11 classifications of chronic primary pain versus chronic secondary musculoskeletal pain versus chronic neuropathic pain.^[14,52,53]^

## 3. Results

### 3.1. Study selection

**Figure 1.**
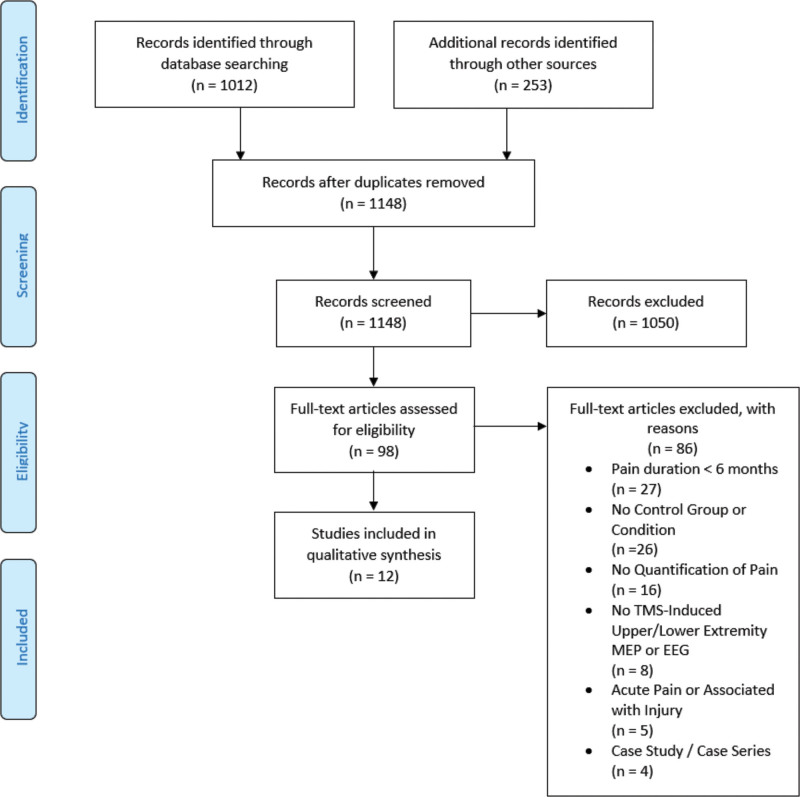
PRISMA (Preferred Reporting Items for Systematic Review and Meta-Analysis) flow chart detailing study flow in literature review.^[41]^

The inclusion and exclusion criteria are illustrated in Figure 1. The search yielded 1012 records, with 253 items from the reference lists. After removing duplicates, 1148 records remained for title and abstract review. Ninety-eight items were retained for full-text review. Twelve articles were included in the data extraction and narrative literature synthesis.

### 3.2. Data extraction

#### 3.2.1. *Participant characteristics*.

Information on the study participants is summarized in Table S1, Supplemental Digital Content, http://links.lww.com/MD/H916. Studies investigated knee osteoarthritis (ICD-11 classification: chronic secondary musculoskeletal pain; 2 studies),^[54,55]^ fibromyalgia (chronic primary pain; 2 studies),^[56,57]^ central post-stroke pain (chronic neuropathic pain; 1 study),^[58]^ diabetic neuropathic pain (chronic neuropathic pain; 1 study)^[59]^ complex regional pain syndrome (chronic primary pain; 2 studies),^[60,61]^ chronic tension-type headache (chronic primary pain; 1 study),^[62]^ chronic shoulder pain (chronic secondary musculoskeletal pain; 1 study),^[63]^ chronic hand pain secondary to central or peripheral nervous lesions (chronic neuropathic pain; 1 study),^[64]^ and phantom limb pain (chronic neuropathic pain; 1 study).^[65]^ Average pain duration across studies was between 1 to 25 years. Eight studies provided details on participants’ baseline disease management, including pharmacotherapy and nutritional supplements.^[54–59,61,64]^

HC participants were included in 9 studies.^[54,56,58–64]^ In total, there were 244 chronic pain patients (192 women; 79%) and 169 HCs (89 women; 53%). The average age of patients with chronic pain and HCs ranged from approximately 35 to 65 years and to 28 to 59 years, respectively. Eight studies were matched for age^[54,56–61,64]^ and sex.^[54,56–58,60–62,64]^

#### 3.2.2. *Study design*.

The details of the study design are presented in Table S2, Supplemental Digital Content, http://links.lww.com/MD/H917. Seven studies used cross-sectional comparisons of chronic pain versus control participants.^[55,56,58–62]^ Five studies reported experimental pain-modulating interventions.^[54,57,63–65]^ Four of the 5 interventional studies were randomized controlled trials^[54,57,64,65]^ and 1 was an open-label study.^[63]^ Interventions included subscapular nerve block versus no intervention for chronic shoulder pain,^[63]^ electrical intramuscular stimulation versus placebo (acupuncture) for knee osteoarthritis,^[54]^ active versus placebo M1 repetitive TMS (rTMS) for chronic hand pain^[64]^ and fibromyalgia,^[57]^ and memantine (glutamate receptor antagonist) versus placebo for phantom limb pain.^[65]^ Intervention durations were 1 session,^[54,63,64]^ 21 days,^[65]^ and 21 weeks.^[57]^ A statistically significant reduction in quantitative pain measurement was achieved in 3 studies:^[54,57,65]^ active versus placebo electrical intramuscular stimulation for knee osteoarthritis,^[54]^ active versus placebo M1 rTMS for fibromyalgia,^[57]^ and memantine and placebo for phantom limb pain.^[65]^

#### 3.2.3. *TMS methods*.

Descriptions of TMS protocols and their putative mechanisms of action are presented in Table 2. The TMS methods used in these studies are listed in Table S3, Supplemental Digital Content, http://links.lww.com/MD/H918. The following TMS protocols were used to determine differences in intracortical and corticospinal electrophysiology either between chronic pain and control participants or pre- and post-intervention: active (active motor threshold [AMT]; 1 study)^[63]^ and (resting motor thresholds [RMT]; 11 studies),^[54–62,64,65]^ (motor evoked potentials [MEPs]; 12 studies),^[54–65]^ (cortical silent period [CSP]; 5 studies),^[54,55,59,63,64]^ (short [SAI]; 3 studies)^[58,60,63]^ and (long-latency afferent inhibition [LAI]; 1 study),^[58]^ (short-interval intracortical inhibition [SICI]; 7 studies),^[54–58,64,65]^ and (intracortical facilitation [ICF]; 7 studies).^[54–58,64,65]^

**Table 2:** Description of TMS paradigms employed in included studies.

TMS protocol	Stimulation characteristics	Neural mechanisms	Studies
***Single pulse***			
Active Motor Threshold (AMT)	Stimulator intensity necessary to elicit MEP with a peak-to-peak amplitude of 100‐200 μV in ≥ 5 of 10 consecutive trials, during sustained tonic contraction of target muscle. Reported as % MSO. Ref:.^[31,33,66,67]^	Characterizes most excitable elements of corticospinal tract. Reflects activity of VGSCs and AMPARs. Influenced by Glu. Ref:.^[35,68]^	Bradnam et al, 2016^[63]^
Cortical Silent Period (CSP)	Quiescence in rectified EMG trace after MEP, when TMS is delivered during sustained tonic contraction of target muscle. Level of contraction and size of MEP do not impact CSP, but CSP duration increases linearly with TMS stimulus intensity. Reported as duration, depth of EMG reduction, or threshold for inhibition. Ref:.^[31,33,69–71]^	Generated by spinal (recurrent inhibition, refractoriness of spinal motor neurons, post-synaptic inhibition) and intracortical (GABA_B_Rs) inhibitory circuits. Influenced by GABA. Ref:.^[35,72–74]^	Bradnam et al, 2016^[63]^ da Graca-Tarragó et al, 2016a^[55]^ da Graca-Tarragó et al, 2016b^[54]^ Lefaucheur et al, 2006^[64]^ Turgut & Altun, 2009^[59]^
Motor Evoked Potential (MEP)	Deflection in EMG trace of target muscle following delivery of threshold or suprathreshold TMS pulse to M1 target area. Measured in active or resting muscle. MEP amplitude increases in sigmoidal relationship with TMS stimulus intensity. Reported as amplitude or elements of MEP-RC, which records MEP amplitude at graduated stimulus intensities. Ref:.^[33,75,76]^	Reflects summation of action potentials in corticospinal axons which synapse with spinal motor neurons. Amplitude reflects corticospinal excitability. MEP-RC reflects gain properties of motor system, representing excitability of multiple populations of motor neurons in corticospinal representation. Influenced by Glu, GABA, 5-HT, and NE. Ref:.^[31,33,35,75–77]^	Bradnam et al, 2016^[63]^ da Graca-Tarragó et al, 2016a^[55]^ da Graca-Tarragó et al, 2016b^[54]^ Lefaucheur et al, 2006^[64]^ Mhalla et al, 2010^[56]^ Mhalla et al, 2011^[57]^ Schwenkreis et al, 2003^[65]^ Tang et al, 2019^[58]^ Turgut & Altun, 2009^[59]^ Turton et al, 2007^[60]^ Vallence et al, 2013^[62]^ van Velzen et al, 2015^[61]^
Resting Motor Threshold (RMT)	Lowest TMS stimulus intensity to elicit MEP with peak-to-peak amplitude of 50 *μ*V in ≥ least 5 of 10 consecutive trials, in resting target muscle. Reported as % MSO. Ref:.^[33,67]^	Characterizes bias level of corticospinal pathway and represents activity of most excitable core of corticospinal and motor neurons. Reflects activity of VGSCs and AMPARs. Influenced by Glu. Ref:.^[35,66,68,75]^	da Graca-Tarragó et al, 2016a^[55]^ da Graca-Tarragó et al, 2016b^[54]^ Lefaucheur et al, 2006^[64]^ Mhalla et al, 2010^[56]^ Mhalla et al, 2011^[57]^ Schwenkreis et al, 2003^[65]^ Tang et al, 2019^[58]^ Turgut & Altun, 2009^[59]^ Turton et al, 2007^[60]^ Vallence et al, 2013^[62]^ van Velzen et al, 2015^[61]^
***Paired pulse***			
Intracortical Facilitation (ICF)	Subthreshold CS followed 10‐15 ms later by suprathreshold TS in relaxed participant. Reported as conditioned MEP normalized to unconditioned MEP. Ref:.^[33,68,73,78]^	Estimate of cortical facilitation by NMDARs, but unclear whether exclusively cortically driven or if there are spinal contributions. Influenced by Glu. Ref:.^[35,73,77,79]^	da Graca-Tarragó et al, 2016a^[55]^ da Graca-Tarragó et al, 2016b^[54]^ Lefaucheur et al, 2006^[64]^ Mhalla et al, 2010^[56]^ Mhalla et al, 2011^[57]^ Schwenkreis et al, 2003^[65]^ Tang et al, 2019^[58]^
Long-latency Afferent Inhibition (LAI)	Electrical CS delivered over peripheral nerve contralateral to M1, followed 100‐1000 ms later by suprathreshold TMS TS over target M1 area in a relaxed participant. Reduction in size of MEP proportionate to intensity of CS. Reported as conditioned MEP normalized to unconditioned MEP. Ref:.^[33,80–83]^	Related to cortical inhibitory mechanisms. Marker of sensorimotor integration, due to assimilation of afferent sensory input in sensorimotor cortex to alter efferent motor output. Mediated by GABA_A_Rs. Influenced by GABA. Ref:.^[35,68,80,82,84–91]^	Tang et al, 2019^[58]^
Short-interval Intracortical Inhibition (SICI)	Subthreshold CS followed 1‐5 ms later by suprathreshold TS in relaxed participant. Magnitude of SICI depends on CS and TS intensity. Reported as conditioned MEP normalized to unconditioned MEP. Ref:.^[31,33,67,78,92,93]^	Cortical phenomenon. Stimuli delivered using 1 ms ISI assess synaptic inhibition modulated by extra-synaptic levels of GABA or by neuronal refractoriness. ISI ≥ 2.5 ms examine GABA_A_R-mediated inhibition. Influenced by GABA. Ref:.^[35,77,94–96]^	da Graca-Tarragó et al, 2016a^[55]^ da Graca-Tarragó et al, 2016b^[54]^ Lefaucheur et al, 2006^[64]^ Mhalla et al, 2010^[56]^ Mhalla et al, 2011^[57]^ Schwenkreis et al, 2003^[65]^ Tang et al, 2019^[58]^
Short-latency Afferent Inhibition (SAI)	Electrical CS delivered over peripheral nerve contralateral to M1, followed 20‐25 ms later by suprathreshold TMS TS over target M1 area in a relaxed participant. Reduction in size of MEP proportionate to intensity of CS. Reported as conditioned MEP normalized to unconditioned MEP. Ref:.^[33,80–83,97]^	Cortical measure involving thalamocortical projections. Marker of sensorimotor integration, due to assimilation of afferent sensory input in sensorimotor cortex to alter efferent motor output. Mediated by GABA_A_Rs and muscarinic Ach receptors. Influenced by GABA and Ach. Ref:.^[35,68,80,82,84–91]^	Bradnam et al, 2016^[63]^ Tang et al, 2019^[58]^ Turton et al, 2007^[60]^

5-HT = serotonin (5-hydroxytryptamine), Ach = acetylcholine, AMPAR = α-amino-3-hydroxy-5-methyl-4-isoxazolepropionic acid receptor (ionotropic transmembrane glutamate receptor), CS = conditioning stimulus, EMG = electromyogram, Glu = glutamate, GABA = γ-aminobutyric acid, GABA_B_R = G-protein coupled γ-aminobutyric acid receptor, GABA_A_R = ionotropic γ-aminobutyric acid receptor (ligand-gated ion channel), ISI = interstimulus interval, M1 = primary motor cortex, MEP = motor evoked potential, MEP-RC = motor evoked potential recruitment curve (stimulus-response curve/ input-output curve), MSO = maximal stimulator output, NE = norepinephrine, NMDAR = N-methyl-D-aspartate receptor (ionotropic glutamate receptor), Ref = references, TMS = transcranial magnetic stimulation, TS = test stimulus, VGSC = voltage-gated sodium channel.

#### 3.2.4. *TMS results*.

The results of the studies are presented in Table 3 and Table S4, Supplemental Digital Content, http://links.lww.com/MD/H919.

**Table 3:** Summary of statistically significant TMS findings by pain ICD-11 pain classification, both compared to healthy control participants and following interventions.

TMS outcome	Neurotransmitter mechanism	Chronic primary pain^1^ (5 studies, ^57,58,61–63^)	Chronic secondary musculoskeletal pain^2^ (3 studies, ^55,56,64^)	Chronic neuropathic pain^3^ (4 studies, ^59,60,65,66^)
AMT/ RMT	AMPARs (Glu); VGSCs	↑ vs HC	↑ vs HC	↓ vs HC
MEP	Glu; GABA; 5-HT; NE	↓ vs HC	↓ after intervention	NS
SICI	GABA_A_Rs (GABA)	↓ vs HC; ↑ after intervention	↑ after intervention	↓ vs HC; ↑/↓ after intervention
ICF	NMDARs (Glu)	↓ vs HC; ↑ after intervention	↓ after intervention	↓ after intervention
CSP	GABA_B_Rs (GABA)	NS	↑ vs HC; ↑/↓ after intervention	↓ vs HC
SAI	Muscarinic Receptors (ACh); GABA_A_Rs (GABA)	—	↑ after intervention	NS
LAI	GABA_A_Rs (GABA)	—	—	NS
Correlation with Clinical Outcomes	—	↑ MEP and ↓ motor imagery; ↑ SICI and ↑ pain catastrophizing; ↓ SICI and ↓ depression; ↓ ICF and ↓ illness impact on function; ↓ ICF and ↓ quality of life; ↓ ICF and ↓ pain catastrophizing	↑ AMT and ↑ pain; ↓ CSP and ↑ pain; ↓ SAI and ↓ pain	↑ SICI and ↓ pain; ↑ SICI and ↓ quality of life; ↑ SICI and ↓ sensory processing; ↓ CSP and ↑ pain

5-HT = serotonin (5-hydroxytryptamine), ACh = acetylcholine, AMT = active motor threshold, AMPAR = α-amino-3-hydroxy-5-methyl-4-isoxazolepropionic acid receptor (ionotropic transmembrane glutamate receptor), CSP = cortical silent period, ICF = intracortical facilitation, ICD-11, International Statistical Classification of Diseases and Related Health Problems, 11^th^ Edition, GABA = γ-aminobutyric acid, GABA_A_R = ionotropic γ-aminobutyric acid receptor (ligand-gated ion channel), GABA_B_R = G-protein coupled γ-aminobutyric acid receptor, Glu = glutamate, HC = healthy control, LAI = long-latency afferent inhibition, MEP = motor evoked potential, NE = norepinephrine, NMDAR = N-methyl-D-aspartate receptor (ionotropic glutamate receptor), NS = not statistically significant at *P <* .05, RMT = resting motor threshold, SAI = short-latency afferent inhibition, SICI = short-interval intracortical inhibition, TMS = transcranial magnetic stimulation, VGSC = voltage-gated sodium channel.

1 Includes complex regional pain syndrome, chronic tension-type headache, and fibromyalgia.^[56,57,60–62]^

2 Includes chronic hand pain, knee osteoarthritis, and chronic shoulder pain.^[54,55,63,64]^

3 Includes central post-stroke pain, diabetic neuropathic pain, and phantom limb pain.^[58,59,65,7,13,14]^

##### 3.2.4.1. *AMT and RMT*.

One study found a statistically significant increase in AMT in the painful infraspinatus muscle of persons with chronic shoulder pain versus the dominant infraspinatus of HCs^[63]^ however, subscapular nerve block had no statistically significant effect on AMT.^[63]^ Three of 11 studies that examined RMT had statistically significant findings.^[55,56,59]^ One study found significantly greater RMT in the first dorsal interosseous muscle (FDI) of persons with knee osteoarthritis versus HCs^[55]^ and another in the bilateral FDI of fibromyalgia patients versus HCs.^[56]^ Conversely, 1 study observed significantly lower RMT in the FDI of patients with diabetic neuropathic pain versus HCs.^[59]^

##### 3.2.4.2. *MEP*.

Four of 12 studies that examined MEPs showed statistically significant findings.^[54,56,61,62]^ Cross-sectionally, 1 study showed significantly smaller MEP amplitude in the relaxed FDI of persons with fibromyalgia versus HCs.^[56]^ In another study, persons with chronic tension-type headache lacked a normal increase in abductor pollicis brevis MEP amplitude that HCs exhibited after skilled motor practice.^[62]^ For interventions, 1 study found that MEP amplitude in the FDI of knee osteoarthritis patients was significantly decreased following electrical intramuscular stimulation of the knee extensors versus placebo.^[54]^

##### 3.2.4.3. *CSP*.

Four of the 5 studies that examined CSP reported statistically significant findings.^[59,63,64]^ Cross-sectionally, 1 study observed a significantly shorter CSP duration in the FDI of persons with diabetic neuropathic pain versus HCs.^[59]^ Similarly, another study reported a significantly shorter CSP duration in the FDI of patients with chronic hand pain versus HCs.^[64]^ In contrast, another study showed that persons with chronic shoulder pain demonstrated a significantly longer CSP duration in the injured infraspinatus muscle versus the dominant infraspinatus of HCs.^[63]^ After subscapular nerve block, CSP became significantly shorter in patients with shoulder pain, closer to that of HCs, but returned to baseline at the 1-week follow-up.^[63]^ Finally, another study observed significantly longer CSP duration in the FDI following active versus placebo electrical intramuscular stimulation in persons with knee osteoarthritis.^[54]^

##### 3.2.4.4. *SAI and LAI*.

One of 3 studies on SAI showed statistically significant findings,^[63]^ whereby subscapular nerve block of the injured infraspinatus muscle in persons with chronic shoulder pain resulted in significantly increased SAI versus pre-intervention.^[63]^ The 1 study employing LAI did not report statistically significant findings.^[58]^

##### 3.2.4.5. *SICI*.

Five of 7 studies on SICI reported statistically significant results.^[56–58,64,65]^ In terms of cross-sectional results, 1 study reported significantly lower SICI in the FDI of fibromyalgia patients versus HCs.^[56]^ In a second study, SICI was significantly lower in the amputated limb of persons with phantom limb pain relative to the FDI, biceps brachii, and deltoid muscles in HCs.^[65]^ In another study, patients with central post-stroke pain had significantly lower SICI in the stroke affected versus unaffected FDI, whereas HCs demonstrated no significant inter-limb difference.^[58]^ Likewise, SICI was significantly lower in the FDI of the painful versus painless hands of patients with chronic hand pain, but not in HCs.^[64]^ From interventions, 2 studies found that active versus placebo M1 rTMS significantly increased SICI values in the FDI of both patients with fibromyalgia^[57]^ and chronic hand pain.^[64]^ Conversely, another study found that memantine, but not placebo, significantly reduced SICI in the amputated limb of persons with phantom limb pain.^[65]^

##### 3.2.4.6. *ICF*.

Five of 7 studies using ICF reported statistically significant outcomes.^[54,56–58,65]^ One cross-sectional study showed significantly lower ICF in the FDI of patients with fibromyalgia versus HCs.^[56]^ In a second study, there was a significantly higher level of ICF in the stroke-affected versus unaffected FDI in patients with central post-stroke pain but not in HCs.^[58]^ In studies employing interventions, increased ICF was found in the dominant FDI of persons with fibromyalgia after active, but not placebo, M1 rTMS.^[57]^ In contrast, a significant decrease in ICF was found in the FDI of persons with knee osteoarthritis after both active and placebo electrical intramuscular stimulation,^[54]^ as well as after memantine versus placebo in the amputated limb of patients with phantom limb pain.^[65]^

#### 3.2.5. *Correlations with clinical outcomes*.

TMS clinical correlations are shown in Table 2. Eleven studies examined the correlation between TMS and clinical outcomes.^[54–59,61–65]^

##### 3.2.5.1. *Quantitative pain measurements*.

Seven studies found statistically significant associations between TMS outcomes and quantitative pain measures.^[55–58,61,63,64]^ In terms of motor thresholds, there was a strong positive relationship between AMT and visual analog scale ratings in patients with chronic shoulder pain.^[63]^ For single-pulse TMS, CSP duration had a strong negative correlation with numerical rating scale scores in participants with knee osteoarthritis^[55]^ and a weak correlation with visual analog scale ratings in participants with chronic hand pain.^[64]^ With reference to paired-pulse TMS, there were statistically significant associations between baseline quantitative pain measures and SAI and SICI.^[57,63,64]^ Baseline SAI had a moderate positive correlation with visual analog scale ratings following subscapular nerve block in patients with chronic shoulder pain.^[63]^ SICI had equivocal findings across studies.^[57,64]^

##### 3.2.5.2. *Secondary clinical outcomes*.

Statistically significant relationships between TMS and secondary clinical outcomes were found in 5 studies.^[55–58,61]^ There were weak-to-moderate associations between TMS outcomes and depression,^[56]^ illness impact on functioning,^[56,57]^ and pain catastrophizing^[56,57]^ in patients with fibromyalgia. There were similar associations between TMS outcomes and quality of life and somatosensory processing in patients with stroke;^[58]^ and with motor imagery in patients with complex regional pain syndrome.^[61]^

#### 3.2.6. *TMS and pain findings by pain classification*.

To generalize our findings and assist in drawing conclusions regarding the neurophysiology of chronic pain, we synthesized statistically significant results across ICD-11 classifications of chronic pain.^[7,13,14]^ The details are summarized in Table 3.

##### 3.2.6.1. *Chronic primary pain*.

Five studies investigated complex regional pain syndrome, chronic tension-type headache, and fibromyalgia.^[56,57,60–62]^ Compared with HCs, there were greater values of RMT and lower values of MEP amplitude, SICI, and ICF. Following the interventions, SICI and ICF tended to decrease to normal values. No TMS measures were related to any quantitative pain measures. However, statistically significant associations were observed between motor imagery (negative correlation with MEP amplitude), depression (positive correlation with SICI), pain catastrophizing (positive correlations with SICI and ICF), illness impact on functioning (positive correlation with ICF), and quality of life (positive correlation with ICF).

##### 3.2.6.2. *Chronic neuropathic pain*.

Four studies examined central post-stroke pain, diabetic neuropathic pain, phantom limb pain, and chronic hand pain secondary to central and peripheral nervous lesions.^[58,59,64,65]^ Relative to the controls, lower values were observed for RMT, SICI, and CSP. After the intervention, the ICF tended to decrease. Both SICI and CSP tended to be negatively associated with quantitative pain measures. Quality of life and sensory processing were negatively correlated with SICI.

##### 3.2.6.3. *Chronic secondary musculoskeletal pain*.

Three studies examined knee osteoarthritis and chronic shoulder pain.^[54,55,63]^ In these studies, AMT, RMT, and CSP tended to be greater in patients than in HCs. After the intervention, SICI and SAI tended to increase, whereas MEP amplitude and ICF tended to decrease. There tended to be positive associations between quantitative pain measures and AMT and SAI and a negative relationship between CSP and pain.

## 4. Discussion

This scoping review aimed to explore the role of TMS as a pathophysiological biomarker for characterization and monitoring of chronic pain. To contextualize our findings, we first provide a brief overview of the neuropathophysiology of chronic pain. Next, we discuss the relevance of TMS indices of M1 excitability as pathophysiological biomarkers for chronic pain.

### 4.1. *Chronic pain neuropathophysiology*

**Figure 2.**
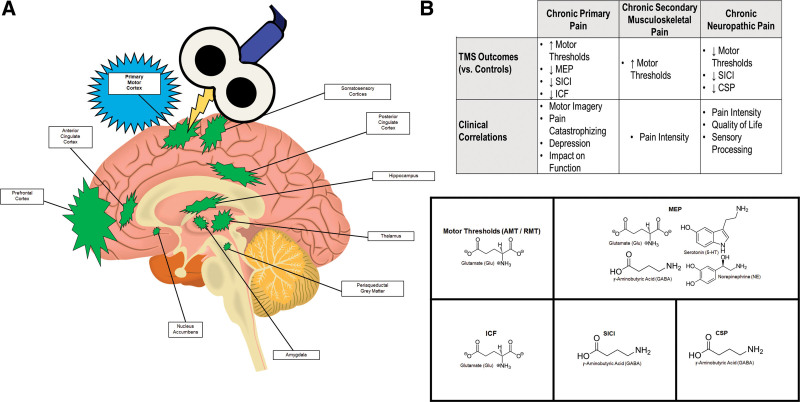
Schematic representation of (A) the “Pain Matrix” network and (B) transcranial magnetic stimulation (TMS) findings related to the primary motor cortex (M1) in chronic noncancer pain. Note: for conciseness, in panel A, the basal ganglia nuclei are not depicted; and in panel B, only cross-sectional TMS findings are reported. CSP = cortical silent period, ICF = intracortical facilitation, MEP = motor evoked potential, SICI = short-interval intracortical inhibition.

The literature describes a coordinated brain network the “pain matrix” (Fig. 2) which modulates pain identification (nociception), suffering in response to pain, and observable pain behaviors.^[10,18,98–103]^ Pain afferents are transmitted the brain via the spinothalamic and other tracts, which originate in the dorsal horn and project to the reticular formation, periaqueductal gray matter, parabrachial nucleus, hypothalamus, and thalamus.^[19,104]^ The reticular formation and periaqueductal gray matter are involved in autonomic and arousal responses, up-/downregulation of pain afferents, and endogenous analgesia.^[10,18,19,100,105–107]^ The parabrachial nucleus projects to the amygdala to increase arousal and physiologic stress^[10,19,100]^ and the hypothalamus regulates neuroendocrine and cardiovascular responses to pain.^[10,18,19,100]^

The thalamus receives nociceptive afferent signals, conveys information related to pain localization, and serves as a gateway to the limbic system, somatosensory cortices, and prefrontal cortex, where attention is drawn to the stimulus, a somatotopic location and qualitative description are assigned, and an affective state and motivation follow the sensation.^[10,18,19,100,103]^ In chronic pain, the thalamus has a reduced ability to regulate incoming pain signals, resulting in the persistence of pain memories and fear behaviors in concert with the hippocampus and amygdala.^[10]^ The cingulate and insular cortices are crucial to the emotional processing of pain, including the integration of sensory, affective, and cognitive aspects of pain, and the autonomic response pain response, including physiological arousal, respectively.^[10,98,108]^ From the thalamus and limbic system are additional projections to the medial and dorsolateral prefrontal cortices, regions involved in emotion regulation and the identification of unpleasantness and dysphoria related to pain.^[10,102,109]^ Additionally, the nucleus accumbens receives inputs from the prefrontal cortex, limbic system, and thalamus, integrates descriptive and affective information about pain, and helps inform motivation and action.^[10,110]^

Two major regions implicated in pain behaviors are the somatosensory cortices and M1.^[98,102,111]^ The somatosensory cortices are significant because of their proposed somatotopic representation of pain, enabling pain identification and localization.^[18,19,98–100,103]^ The premotor cortex and M1 serve as association cortices to combine attentional, cognitive, and emotional information to monitor and execute motor activities in response to pain.^[112,113]^ The motor and somatosensory cortices demonstrate attenuated control over motor output in during pain, partly due to changes in excitability and inhibition in the presence of painful stimuli.^[102,113–115]^ Chronic pain has a detrimental impact on motor learning^[111,116,117]^ and M1 structure and function.^[102,111,114]^

Reciprocal connections between M1 and the somatosensory cortices suggest a bidirectional relationship between pain perception and pain behavior, whereby pain signals influence motor output, whereas deliberate movement (e.g., pain behaviors) and M1 neuromodulation affect pain representation in the somatosensory cortices.^[102,111,116,117]^

### 4.2. TMS in chronic pain

Based on TMS evidence of chronic pain associations with abnormal M1 excitability, changes in TMS outcomes accompanied by pain reduction, and reductions in pain ratings with M1 neurostimulation, M1 may be critical for understanding chronic pain.^[102,111,114]^ Although there is likely no single universal common pathway for all chronic pain disorders, our findings suggest that TMS may enhance the understanding of chronic pain neuropathophysiology by probing M1 changes in the context of specific pain etiologies (e.g., chronic primary pain, chronic neuropathic pain, and chronic secondary musculoskeletal pain). The literature indicates that, TMS is useful for indexing circuits related to glutamate (AMT, RMT, MEP, ICF), GABA (MEP, SICI, CSP, SAI, LAI), acetylcholine (SAI), voltage-gated sodium channels (AMT and RMT), serotonin (MEP), and norepinephrine (MEP).^[24,35–37]^

#### 4.2.1. *Chronic primary pain*.

Chronic primary pain is characterized by its elusive pathophysiology, difficult localization, interaction with biopsychosocial factors, and negative impact on mood and function.^[53,118]^ Studies of chronic primary pain neuropathophysiology show aberrant structure and function in brain areas, such as the thalamus, limbic system, prefrontal cortices.^[118–128]^

We observed a tendency for motor thresholds to be higher and MEP amplitude, SICI, and ICF to be lower in patients with chronic primary pain than in HCs.^[56,57,60–62]^ After interventions, both SICI and ICF tended to increase to normal levels. No TMS outcomes were related to quantitative pain measures; however, there were positive correlations between pain catastrophizing and SICI and ICF, depression and SICI, and ICF and illness impact on functioning and quality of life. These findings were suggestive of aberrant intracortical GABAergic inhibition and glutamatergic facilitation,^[35]^ which are related to elements of suffering and function, as opposed to pain perception.

#### 4.2.2. *Chronic neuropathic pain*.

Chronic neuropathic pain is caused by lesions or disease of the central or peripheral somatosensory nervous systems with abnormal sensory symptoms in the somatotopic territory of the affected nervous structure.^[14]^ Central sensitization in dorsal horn neurons is a main pathophysiological mechanism, especially in peripheral chronic neuropathic pain,^[129]^ while damage affecting the thalamus or spinothalamic tract is involved in central neuropathic pain.^[19,119,121]^ In chronic neuropathic pain, there is evidence of deficient GABAergic and excessive glutamatergic activity.^[129]^ Further work suggests that insufficient monoamine signaling in the CNS prevents adequate descending inhibition of pain.^[130]^

We observed that motor thresholds, SICI, and CSP tended to be lower in patients with chronic neuropathic pain than in controls.^[58,59,64,65]^ Post-intervention, the ICF tended to decrease. Correlations were found between pain quantification and SICI and CSP, whereas SICI was related to quality of life and somatosensory function. Our findings support the literature, suggesting reduced GABAergic activity in chronic neuropathic pain. While there was no evidence of excessive glutamatergic activity cross-sectionally, we observed reductions in intracortical glutamatergic facilitation (ICF) after the intervention.

#### 4.2.3. *Chronic secondary musculoskeletal pain*.

Chronic secondary musculoskeletal pain arises from underlying diseases of the musculoskeletal system secondary to inflammatory processes (e.g., infection, crystal formation, autoimmunity, mechanical degeneration) or neurologic diseases (e.g., spasticity and hypertonicity).^[52]^ Chronic secondary musculoskeletal pain appears to be related to dysfunction in the monoamine and catecholamine neurotransmitter pathways,^[131]^ as well as glutamate and GABA systems.^[17]^

We found mixed evidence for TMS markers of neurotransmitter activity.^[54,55,63]^ Cross-sectionally, both motor thresholds and CSP tended to be greater in patients with chronic secondary musculoskeletal pain than in HCs. After the interventions, there tended to be increases in SICI and SAI and decreases in MEP amplitude and ICF. There tended to be positive correlations between quantitative pain ratings and AMT and SAI and an inverse correlation between CSP and pain quantification. Our findings suggest that low glutamatergic and GABAergic activity is related to chronic secondary musculoskeletal pain. Further increases in GABAergic inhibition and decreases in glutamatergic excitability and facilitation were related to improvements in pain.

### 4.3. *Limitations*

This study had several limitations. First, owing to our search strategy, we evaluated a small, but representative group of articles on chronic pain. We restricted our search to a single database and included studies on pain lasting for at least 6 months. However, by restricting our search criteria and grouping studies according to the ICD-11 classifications, we stratified groups of relatively homogeneous studies and qualitatively examined the evidence in this context. In comparison, a previous meta-analysis of TMS in chronic pain used a threshold pain duration of at least 3 months and yielded 35 studies that were heterogeneous in terms of pain duration and etiology^.[114]^ The authors found no statistically significant effect of chronic pain on any TMS outcome.^[114]^ Using our methods, we were able to qualitatively discern unique findings for each chronic pain etiology that may have been washed out if studies were not stratified accordingly. This information can inform future systematic reviews or meta-analyses to approach the evidence.

Second, because we performed a scoping review, no critical appraisal exercises were performed. Consequently, experimental or publication bias in these studies may have led to false conclusions. However, we aimed to provide exhaustive information about the study characteristics and extensive summaries of the study results. We believe that readers have the necessary information to appraise our findings critically.

Finally, we acknowledge that stratifying chronic pain syndromes using the ICD-11 diagnoses may be viewed by some readers as problematic. Given that several studies have predated ICD-11, we may have misattributed pain etiologies in our synthesis, potentially leading to false conclusions. Nevertheless, we carefully examined the studies’ diagnostic criteria and cross-referenced ICD-11 to be as accurate as possible. Our approach is rigorous and has heightened validity compared with discussing chronic pain in general, without distinguishing the underlying etiology.

## 5. Conclusions

We conducted a scoping review to explore the role of TMS as a pathophysiological biomarker for characterizing and monitoring chronic non-cancer pain. We discuss our findings within the context of chronic pain neuropathophysiology, with special reference to the “pain matrix.” We observed that abnormalities in GABAergic and glutamatergic circuits tended to underlie chronic pain; however, the direction (i.e., increased or decreased versus HCs), locus of change (i.e., corticospinal versus intracortical), response to interventions, and relationships with clinical outcomes were unique to each chronic pain etiology. Given the distinct findings observed across different chronic pain etiologies, this strategy could serve as a template for future studies to better understand chronic pain neurophysiology.

## Author contributions

**Conceptualization:** Nicholas Jacob Snow, Megan Christine Kirkland, Matthew Bruce Downer, Hannah Margaret Murphy, Michelle Ploughman.

**Data curation:** Nicholas Jacob Snow.

**Formal analysis:** Nicholas Jacob Snow, Megan Christine Kirkland, Matthew Bruce Downer, Hannah Margaret Murphy.

**Methodology:** Nicholas Jacob Snow, Megan Christine Kirkland, Matthew Bruce Downer, Michelle Ploughman.

**Supervision:** Michelle Ploughman.

**Writing – original draft:** Nicholas Jacob Snow, Megan Christine Kirkland, Matthew Bruce Downer, Hannah Margaret Murphy, Michelle Ploughman.

**Writing – review & editing:** Nicholas Jacob Snow, Megan Christine Kirkland, Matthew Bruce Downer, Hannah Margaret Murphy, Michelle Ploughman.

## Supplementary Material

## References

[1] Reid KJ Harker J Bala MM. Epidemiology of chronic non-cancer pain in Europe: Narrative review of prevalence, pain treatments and pain impact. Curr Med Res Opin. 2011;27:449–62. 21194394 10.1185/03007995.2010.545813

[2] Schopflocher D Taenzer P Jovey R. The prevalence of chronic pain in Canada. Pain Res Manag. 2011;16:445–50. 22184555 10.1155/2011/876306 PMC3298051

[3] Institute of Medicine (IOM). Relieving Pain in America: A Blueprint for Transforming Prevention, Care, Education, and Research. Washington, DC: National Academies Press, 2011:55–112. 22553896

[4] Goldberg DS McGee SJ. Pain as a global public health priority. BMC Public Health. 2011;11:770. 21978149 10.1186/1471-2458-11-770 PMC3201926

[5] Mäntyselkä P Kumpusalo E Ahonen R. Pain as a reason to visit the doctor: a study in Finnish primary health care. Pain. 2001;89:175–80. 11166473 10.1016/s0304-3959(00)00361-4

[6] Tauben D Stacey BR. Evaluation of chronic non-cancer pain in adults. In: Post TW, (ed). UpToDate. Waltham, MA, USA: UpToDate, Inc., 2020. Available at: https://www.uptodate.com/contents/evaluation-of-chronicnon-cancer-pain-in-adults?search=Evaluation%20of%20chronic%20noncancer%20pain%20in%20adults.&source=search_result&selectedTitle=1~150&usage_type=default&display_rank=1 [November 14, 2022].

[7] Treede R-D Rief W Barke A. A classification of chronic pain for ICD-11. Pain. 2015;156:1003–7. 25844555 10.1097/j.pain.0000000000000160 PMC4450869

[8] Cervero F. Visceral pain - Central sensitisation. Gut. 2000;47(Suppl. 4):56iv56–57. 10.1136/gut.47.suppl_4.iv56 PMC1766826 11076916

[9] Mills SEE Nicolson KP Smith BH. Chronic pain: a review of its epidemiology and associated factors in population-based studies. Br J Anaesth. 2019;123:e273–83. 31079836 10.1016/j.bja.2019.03.023 PMC6676152

[10] Yang S Chang MC. Chronic pain: structural and functional changes in brain structures and associated negative affective states. Int J Mol Sci. 2019;20:3130. 10.3390/ijms20133130 PMC6650904 31248061

[11] Wu Y Yao X Jiang Y. Pain aversion and anxiety-like behavior occur at different times during the course of chronic inflammatory pain in rats. J Pain Res. 2017:2585–2593. 29158690 10.2147/JPR.S139679 PMC5683785

[12] Crofford LJ. Chronic pain: where the body meets the brain. Trans Am Clin Climatol Assoc. 2015;126:167–83. 26330672 PMC4530716

[13] World Health Organization (WHO). International Statistical Classification of Diseases and Related Health Problems. 11th ed.; 2019. Available at: https://icd.who.int/browse11 [November 14, 2022].

[14] Scholz J Finnerup NB Attal N. The IASP classification of chronic pain for ICD-11: chronic neuropathic pain. Pain. 2019;160:53–9. 30586071 10.1097/j.pain.0000000000001365 PMC6310153

[15] Gerdle B Ghafouri B Ernberg M. Chronic musculoskeletal pain: review of mechanisms and biochemical biomarkers as assessed by the microdialysis technique. J Pain Res. 2014;7:313–26. 24966693 10.2147/JPR.S59144 PMC4062547

[16] Hansson E Rönnbäck L. Altered neuronal-glial signaling in glutamatergic transmission as a unifying mechanism in chronic pain and mental fatigue. Neurochem Res. 2004;29:989–96. 15139297 10.1023/b:nere.0000021243.86287.43

[17] Peek AL Rebbeck T Puts NA. Brain GABA and glutamate levels across pain conditions: a systematic literature review and meta-analysis of 1H-MRS studies using the MRS-Q quality assessment tool. Neuroimage. 2020;210:116532. 31958584 10.1016/j.neuroimage.2020.116532

[18] Baker K. Recent advances in the neurophysiology of chronic pain. EMA Emerg Med Australas. 2005;17:65–72. 15675907 10.1111/j.1742-6723.2005.00689.x

[19] Basbaum AI Jessell TM. Pain. Kandel ER Schwartz JH Jessell TM Siegelbaum SA Hudspeth AJ, (eds). In: Principles of Neural Science. 5th ed. New York, NY: McGraw-Hill, 2013:530–555.

[20] Khan N Smith MT. Multiple sclerosis-induced neuropathic pain: Pharmacological management and pathophysiological insights from rodent EAE models. Inflammopharmacology. 2014;22:1–22. 24234347 10.1007/s10787-013-0195-3 PMC3933737

[21] Iannitti T Kerr BJ Taylor BK. Mechanisms and pharmacology of neuropathic pain in multiple sclerosis. Curr Top Behav Neurosci. 2014;20:75–97. 24590824 10.1007/7854_2014_288 PMC4464806

[22] Herman RM D’Luzansky SC Ippolito R. Intrathecal baclofen suppresses central pain in patients with spinal lesions. A pilot study. Clin J Pain. 1992;8:338–45. 1493344

[23] O’Connor AB Schwid SR Herrmann DN. Pain associated with multiple sclerosis: systematic review and proposed classification. Pain. 2008;137:96–111. 17928147 10.1016/j.pain.2007.08.024

[24] Barr MS Farzan F Davis KD. Measuring GAB aergic inhibitory activity with TMS-EEG and its potential clinical application for chronic pain. J Neuroimmune Pharmacol. 2013;8:535–46. 22744222 10.1007/s11481-012-9383-y

[25] Solaro C Boehmker M Tanganelli P. Pregabalin for treating paroxysmal painful symptoms in multiple sclerosis: a pilot study. J Neurol. 2009;256:1773–4. 19579001 10.1007/s00415-009-5203-6

[26] Solaro C Lunardi GL Capello E. An open-label trial of gabapentin treatment of paroxysmal symptoms in multiple sclerosis patients. Neurology. 1998;51:609–11. 9710049 10.1212/wnl.51.2.609

[27] Houtchens MK Richert JR Sami A. Open label gabapentin treatment for pain in multiple sclerosis. Mult Scler. 1997;3:250–3. 9372509 10.1177/135245859700300407

[28] Szok D Tajti J Nyári A. Therapeutic approaches for peripheral and central neuropathic pain. Behav Neurol. 2019;2019:1–13. 10.1155/2019/8685954 PMC6906810 31871494

[29] Biomarkers Definitions Working Group. Biomarkers and surrogate endpoints: preferred definitions and conceptual framework. Clin Pharmacol Ther. 2001;69:89–95. 11240971 10.1067/mcp.2001.113989

[30] Thibaut A Zeng D Caumo W. Corticospinal excitability as a biomarker of myofascial pain syndrome. Pain Reports. 2017;2:e5941–8. 10.1097/PR9.0000000000000594 PMC5741300 29392210

[31] Groppa S Oliviero A Eisen A. A practical guide to diagnostic transcranial magnetic stimulation: report of an IFCN committee. Clin Neurophysiol. 2012;123:858–82. 22349304 10.1016/j.clinph.2012.01.010 PMC4890546

[32] Lefaucheur JP Aleman A Baeken C. Evidence-based guidelines on the therapeutic use of repetitive transcranial magnetic stimulation (rTMS): an update (2014–2018). Clin Neurophysiol. 2020;131:474–528. 31901449 10.1016/j.clinph.2019.11.002

[33] Rossini PM Burke D Chen R. Non-invasive electrical and magnetic stimulation of the brain, spinal cord, roots and peripheral nerves: basic principles and procedures for routine clinical and research application. An updated report from an I.F.C.N. Committee. Clin Neurophysiol. 2015;126:1071–107. 25797650 10.1016/j.clinph.2015.02.001 PMC6350257

[34] Barker AT Jalinous R Freeston IL. Non-invasive magnetic stimulation of human motor cortex. Lancet. 1985;1:1106–7. 2860322 10.1016/s0140-6736(85)92413-4

[35] Ziemann U Reis J Schwenkreis P. TMS and drugs revisited 2014. Clin Neurophysiol. 2015;126:1847–68. 25534482 10.1016/j.clinph.2014.08.028

[36] Premoli I Castellanos N Rivolta D. TMS-EEG signatures of GABAergic neurotransmission in the human cortex. J Neurosci. 2014;34:5603–12. 24741050 10.1523/JNEUROSCI.5089-13.2014 PMC6608220

[37] Hui J Zomorrodi R Lioumis P. Pharmacological mechanisms of interhemispheric signal propagation: a TMS-EEG study. Neuropsychopharmacology. 2020;45:932–9. 31357206 10.1038/s41386-019-0468-7 PMC7162860

[38] Shamseer L Moher D Clarke M. Preferred reporting items for systematic review and meta-analysis protocols (PRISMA-P) 2015: elaboration and explanation. BMJ. 2015;349:g76471–g7647. 10.1136/bmj.g7647 25555855

[39] Moher D Shamseer L Clarke M. Preferred reporting items for systematic reviews and meta-analysis protocols (PRISMA-P) 2015 statement. Syst Rev. 2015;4:1. 25554246 10.1186/2046-4053-4-1 PMC4320440

[40] Tricco AC Lillie E Zarin W. PRISMA extension for scoping reviews (PRISMA-ScR): checklist and explanation. Ann Intern Med. 2018;169:467–73. 30178033 10.7326/M18-0850

[41] Moher D Liberati A Tetzlaff J. Preferred reporting items for systematic reviews and meta-analyses: the PRISMA statement. PLoS Med. 2009;6:e1000097. 19621072 10.1371/journal.pmed.1000097 PMC2707599

[42] Arksey H O’Malley L. Scoping studies: towards a methodological framework. Int J Soc Res Methodol Theory Pract. 2005;8:19–32.

[43] Levac D Colquhoun H O’Brien KK. Scoping studies: advancing the methodology. Implement Sci 2010;5:69. 20854677 10.1186/1748-5908-5-69 PMC2954944

[44] Campbell M Katikireddi SV Sowden A. Lack of transparency in reporting narrative synthesis of quantitative data: a methodological assessment of systematic reviews. J Clin Epidemiol. 2019;105:1–9. 30196129 10.1016/j.jclinepi.2018.08.019 PMC6327109

[45] Campbell M McKenzie JE Sowden A. Synthesis without meta-analysis (SWiM) in systematic reviews: reporting guideline. BMJ. 2020;368:1–6. 10.1136/bmj.l6890 PMC7190266 31948937

[46] Van der Mierden S Tsaioun K Bleich A. Software tools for literature screening in systematic reviews in biomedical research. ALTEX. 2019;36:508–17. 31113000 10.14573/altex.1902131

[47] Schardt C Adams MB Owens T. Utilization of the PICO framework to improve searching PubMed for clinical questions. BMC Med Inform Decis Mak. 2007;7:1–6. 17573961 10.1186/1472-6947-7-16 PMC1904193

[48] Daudt HML Van Mossel C Scott SJ. Enhancing the scoping study methodology: a large, inter-professional team’s experience with Arksey and O’Malley’s framework. BMC Med Res Methodol. 2013;13:1–9. 23522333 10.1186/1471-2288-13-48 PMC3614526

[49] Kim J Shin W. How to do random allocation (randomization). Clin Orthop Surg. 2014;6:103–9. 24605197 10.4055/cios.2014.6.1.103 PMC3942596

[50] Institute of Medicine (IOM). Eden J Levit L Berg A Morton S, (eds). In: Finding What Works in Health Care: Standards for Systematic Reviews. Washington, DC: National Academy Press, 2011:155–94. 24983062

[51] Popay J Roberts H Sowden A. Guidance on the conduct of narrative synthesis in systematic reviews, A Product from the ESRC Methods Programme. Version 1 2006.

[52] Perrot S Cohen M Barke A. The IASP classification of chronic pain for ICD-11: chronic secondary musculoskeletal pain. Pain. 2019;160:77–82. 30586074 10.1097/j.pain.0000000000001389

[53] Nicholas M Vlaeyen JWS Rief W. The IASP classification of chronic pain for ICD-11: chronic primary pain. Pain. 2019;160:28–37. 30586068 10.1097/j.pain.0000000000001390

[54] da Graca-Tarragó ML Deitos A Brietzke AP. Electrical intramuscular stimulation in osteoarthritis enhances the inhibitory systems in pain processing at cortical and cortical spinal system. Pain Med (United States) 2016;17:877–91. 10.1111/pme.12930 26398594

[55] da Graca Tarragó ML Deitos A Brietzke AP. Descending control of nociceptive processing in knee osteoarthritis is associated with intracortical disinhibition. Med (United States) 2016;95:e3353. 10.1097/MD.0000000000003353 PMC4998685 27124022

[56] Mhalla A de Andrade DC Baudic S. Alteration of cortical excitability in patients with fibromyalgia. Pain. 2010;149:495–500. 20356675 10.1016/j.pain.2010.03.009

[57] Mhalla A Baudic S De Andrade DC. Long-term maintenance of the analgesic effects of transcranial magnetic stimulation in fibromyalgia. Pain. 2011;152:1478–85. 21397400 10.1016/j.pain.2011.01.034

[58] Tang SC Lee LJH Jeng JS. Pathophysiology of central poststroke pain motor cortex disinhibition and its clinical and sensory correlates. Stroke. 2019;50:2851–7. 31500556 10.1161/STROKEAHA.119.025692

[59] Turgut N Altun BU. Cortical disinhibition in diabetic patients with neuropathic pain. Acta Neurol Scand. 2009;120:383–8. 19922582 10.1111/j.1600-0404.2009.01235.x

[60] Turton AJ McCabe CS Harris N. Sensorimotor integration in complex regional pain syndrome: a transcranial magnetic stimulation study. Pain. 2007;127:270–5. 17011705 10.1016/j.pain.2006.08.021

[61] Van Velzen GAJ Marinus J Van Dijk JG. Motor cortical activity during motor tasks is normal in patients with complex regional pain syndrome. J Pain. 2015;16:87–94. 25451624 10.1016/j.jpain.2014.10.010

[62] Vallence AM Smith A Tabor A. Chronic tension-type headache is associated with impaired motor learning. Cephalalgia. 2013;33:1048–54. 23598373 10.1177/0333102413483932

[63] Bradnam L Shanahan EM Hendy K. Afferent inhibition and cortical silent periods in shoulder primary motor cortex and effect of a suprascapular nerve block in people experiencing chronic shoulder pain. Clin Neurophysiol. 2016;127:769–78. 25900020 10.1016/j.clinph.2015.03.012

[64] Lefaucheur JP Drouot X Ménard-Lefaucheur I. Motor cortex rTMS restores defective intracortical inhibition in chronic neuropathic pain. Neurology. 2006;67:1568–74. 17101886 10.1212/01.wnl.0000242731.10074.3c

[65] Schwenkreis P Maier C Pleger B. NMDA-mediated mechanisms in cortical excitability changes after limb amputation. Acta Neurol Scand. 2003;108:179–84. 12911461 10.1034/j.1600-0404.2003.00114.x

[66] Capaday C. Neurophysiological methods for studies of the motor system in freely moving human subjects. J Neurosci Methods. 1997;74:201–18. 9219889 10.1016/s0165-0270(97)02250-4

[67] Rossini PM Barker AT Berardelli A. Non-invasive electrical and magnetic stimulation of the brain, spinal cord and roots: basic principles and procedures for routine clinical application. Report of an IFCN committee. Electroencephalogr Clin Neurophysiol. 1994;91:79–92. 7519144 10.1016/0013-4694(94)90029-9

[68] Brown KE Neva JL Ledwell NM. Use of transcranial magnetic stimulation in the treatment of selected movement disorders. Degener Neurol Neuromuscul Dis. 2014;4:133. 32669907 10.2147/DNND.S70079 PMC7337234

[69] Cantello R Gianelli M Civardi C. Magnetic brain stimulation: the silent period after the motor evoked potential. Neurology. 1992;42:1951–9. 1407578 10.1212/wnl.42.10.1951

[70] Merton PA Morton HB. Stimulation of the cerebral cortex in the intact human subject. Nature. 1980;285:227227 227. 10.1038/285227a0 7374773

[71] Inghilleri M Berardelli A Cruccu G. Silent period evoked by transcranial stimulation of the human cortex and cervicomedullary junction. J Physiol. 1993;466:521–34. 8410704 PMC1175490

[72] Chen R Lozano AM Ashby P. Mechanism of the silent period following transcranial magnetic stimulation. Exp Brain Res. 1999;128:539–42. 10541749 10.1007/s002210050878

[73] Chen R Cros D Curra A. The clinical diagnostic utility of transcranial magnetic stimulation: report of an IFCN committee. Clin Neurophysiol. 2008;119:504–32. 18063409 10.1016/j.clinph.2007.10.014

[74] Yacyshyn AF Woo EJ Price MC. Motoneuron responsiveness to corticospinal tract stimulation during the silent period induced by transcranial magnetic stimulation. Exp Brain Res. 2016;234:3457–63. 27481287 10.1007/s00221-016-4742-1

[75] Devanne H Lavoie BA Capaday C. Input–output properties and gain changes in the human corticospinal pathway. Exp Brain Res. 1997;114:329–38. 9166922 10.1007/pl00005641

[76] Ridding MC Rothwell JC. Stimulus/response curves as a method of measuring motor cortical excitability in man. Electroencephalogr Clin Neurophysiol. 1997;105:340–4. 9362997 10.1016/s0924-980x(97)00041-6

[77] Di Lazzaro V Profice P Ranieri F. I-wave origin and modulation. Brain Stimul. 2012;5:512–25. 21962980 10.1016/j.brs.2011.07.008

[78] Kujirai T Caramia MD Rothwell JC. Corticocortical inhibition in human motor cortex. J Physiol. 1993;471:501–19. 8120818 10.1113/jphysiol.1993.sp019912 PMC1143973

[79] Ziemann U Rothwell JC Ridding MC. Interaction between intracortical inhibition and facilitation in human motor cortex. J Physiol. 1996;496:873–81. 8930851 10.1113/jphysiol.1996.sp021734 PMC1160871

[80] Chen R Corwell B Hallett M. Modulation of motor cortex excitability by median nerve and digit stimulation. Exp Brain Res. 1999;129:77–86. 10550505 10.1007/s002210050938

[81] Devanne H Degardin A Tyvaert L. Afferent-induced facilitation of primary motor cortex excitability in the region controlling hand muscles in humans. Eur J Neurosci. 2009;30:439–48. 19686433 10.1111/j.1460-9568.2009.06815.x

[82] Tokimura H Di Lazzaro V Tokimura Y. Short latency inhibition of human hand motor cortex by somatosensory input from the hand. J Physiol. 2000;523:503–13. 10699092 10.1111/j.1469-7793.2000.t01-1-00503.x PMC2269813

[83] Delwaide PJ Olivier E. Conditioning transcranial cortical stimulation (TCCS) by exteroceptive stimulation in parkinsonian patients. Adv Neurol. 1990;53:175–81. 2239457

[84] Turco CV El-Sayes J Locke MB. Effects of lorazepam and baclofen on short- and long-latency afferent inhibition. J Physiol. 2018;596:5267–80. 30192388 10.1113/JP276710 PMC6209752

[85] Turco CV Toepp SL Foglia SD. Association of short- and long-latency afferent inhibition with human behavior. Clin Neurophysiol. 2021;132:1462–80. 34030051 10.1016/j.clinph.2021.02.402

[86] Bailey AZ Asmussen MJ Nelson AJ. Short-latency afferent inhibition determined by the sensory afferent volley. J Neurophysiol. 2016;116:637–44. 27226451 10.1152/jn.00276.2016 PMC4982902

[87] Asmussen MJ Jacobs MF Lee KGH. Short-latency afferent inhibition modulation during finger movement. PLoS One. 2013;8:e60496. 23593228 10.1371/journal.pone.0060496 PMC3617156

[88] Turco CV El-Sayes J Fassett HJ. Modulation of long-latency afferent inhibition by the amplitude of sensory afferent volley. J Neurophysiol. 2017;118:610–8. 28446579 10.1152/jn.00118.2017 PMC5511865

[89] Di Lazzaro V Pilato F Dileone M. Dissociated effects of diazepam and lorazepam on short-latency afferent inhibition. J Physiol. 2005;569:315–23. 16141274 10.1113/jphysiol.2005.092155 PMC1464195

[90] Di Lazzaro V Oliviero A Saturno E. Effects of lorazepam on short latency afferent inhibition and short latency intracortical inhibition in humans. J Physiol. 2005;564:661–8. 15718269 10.1113/jphysiol.2004.061747 PMC1464438

[91] Di Lazzaro V Pilato F Dileone M. Segregating 2 inhibitory circuits in human motor cortex at the level of GABAA receptor subtypes: a TMS study. Clin Neurophysiol. 2007;118:2207–14. 17709293 10.1016/j.clinph.2007.07.005

[92] Di Lazzaro V Restuccia D Oliviero A. Magnetic transcranial stimulation at intensities below active motor threshold activates intracortical inhibitory circuits. Exp Brain Res. 1998;119:265–8. 9535577 10.1007/s002210050341

[93] Sanger T Garg R Chen R. Interactions between 2 different inhibitory systems in the human motor cortex. J Physiol. 2001;530:307–17. 11208978 10.1111/j.1469-7793.2001.0307l.x PMC2278414

[94] Nakamura H Kitagawa H Kawaguchi Y. Intracortical facilitation and inhibition after transcranial magnetic stimulation in conscious humans. J Physiol. 1997;498:817–23. 9051592 10.1113/jphysiol.1997.sp021905 PMC1159197

[95] Stagg CJ. Magnetic resonance spectroscopy as a tool to study the role of GABA in motor-cortical plasticity. Neuroimage. 2014;86:19–27. 23333699 10.1016/j.neuroimage.2013.01.009

[96] Hanajima R Furubayashi T Iwata NK. Further evidence to support different mechanisms underlying intracortical inhibition of the motor cortex. Exp Brain Res. 2003;151:427–34. 12830341 10.1007/s00221-003-1455-z

[97] Mariorenzi R Zarola F Caramia MD. Non-invasive evaluation of central motor tract excitability changes following peripheral nerve stimulation in healthy humans. Electroencephalogr Clin Neurophysiol Pot Sect. 1991;81:90–101. 10.1016/0168-5597(91)90002-f 1708719

[98] Jones A. The pain matrix and neuropathic pain. Brain. 1998;121:783–4. 9619184 10.1093/brain/121.5.783

[99] Loeser JD Melzack R. Pain: An overview. Lancet 1999;353:1607–9. 10334273 10.1016/S0140-6736(99)01311-2

[100] Tracey I. Imaging pain. Br J Anaesth. 2008;101:32–9. 18556697 10.1093/bja/aen102

[101] Damascelli M Woodward TS Sanford N. Multiple functional brain networks related to pain perception revealed by fMRI. Neuroinformatics. 2021;13:1268. 10.1007/s12021-021-09527-6 PMC9537130 34101115

[102] Zaghi S Thiele B Pimentel D. Assessment and treatment of pain with non-invasive cortical stimulation. Restor Neurol Neurosci. 2011;29:439–51. 22124038 10.3233/RNN-2011-0615

[103] Sandkuhler J Bromm B Gebhart GF. Nervous system plasticity and chronic pain. Prog Brain Res. 2000;129:3–543.11098678

[104] Willis WD. Nociceptive pathways: anatomy and physiology of nociceptive ascending pathways. Philos Trans R Soc London Ser B Biol Sci. 1985;308:253–70. 2858882 10.1098/rstb.1985.0025

[105] Martins I Tavares I. Reticular formation and pain: the past and the future. Front Neuroanat. 2017;11:1–14. 28725185 10.3389/fnana.2017.00051 PMC5497058

[106] Urban M Gebhart G. Central mechanisms in pain. Med Clin North Am. 1999;83:585–96. 10386116 10.1016/s0025-7125(05)70125-5

[107] Boureau F Luu M Doubrere J. Study of experimental pain measures and nociceptive reflex in chronic pain patients and normal subjects. Pain. 1991;44:131–8. 2052379 10.1016/0304-3959(91)90126-I

[108] Brown CA Seymour B El-Deredy W. Confidence in beliefs about pain predicts expectancy effects on pain perception and anticipatory processing in right anterior insula. Pain. 2008;139:324–32. 18584963 10.1016/j.pain.2008.04.028

[109] Ong WY Stohler CS Herr DR. Role of the prefrontal cortex in pain processing. Mol Neurobiol. 2019;56:1137–66. 29876878 10.1007/s12035-018-1130-9 PMC6400876

[110] McCarberg B Peppin J. Pain pathways and nervous system plasticity: learning and memory in pain. Pain Med. 2019;20:2421–37. 30865778 10.1093/pm/pnz017

[111] Holmes SA Kim A Borsook D. The brain and behavioral correlates of motor-related analgesia (MRA). Neurobiol Dis. 2021;148:105158. 33157210 10.1016/j.nbd.2020.105158

[112] Leite J Carvalho S Battistella LR. The role of primary motor cortex as a marker and modulator of pain processing and emotional-affective processing. Front Hum Neurosci. 2017;11:270. 28588468 10.3389/fnhum.2017.00270 PMC5440504

[113] Wang W Ho RLM Gatto B. Cortical dynamics of movement-evoked pain in chronic low back pain. J Physiol. 2021;599:289–305. 33067807 10.1113/JP280735

[114] Chang WJ O’Connell NE Beckenkamp PR. Altered primary motor cortex structure, organization, and function in chronic pain: a systematic review and meta-analysis. J Pain. 2018;19:341–59. 29155209 10.1016/j.jpain.2017.10.007

[115] Seminowicz DA Davis KD. Cortical responses to pain in healthy individuals depends on pain catastrophizing. Pain. 2006;120:297–306. 16427738 10.1016/j.pain.2005.11.008

[116] Nijs J Daenen L Cras P. Nociception affects motor output: a review on sensory-motor interaction with focus on clinical implications. Clin J Pain. 2012;28:175–81. 21712714 10.1097/AJP.0b013e318225daf3

[117] Hodges PW. Pain and motor control: from the laboratory to rehabilitation. J Electromyogr Kinesiol. 2011;21:220–8. 21306915 10.1016/j.jelekin.2011.01.002

[118] Sluka KA Clauw DJ. Neurobiology of fibromyalgia and chronic widespread pain. Neuroscience. 2016;338:114–29. 27291641 10.1016/j.neuroscience.2016.06.006 PMC5083139

[119] Marinus J Moseley L Birklein F. Clinical features and pathophysiology of Complex Regional Pain Syndrome – current state of the art. Lancet Neurol. 2011;10:637–48. 21683929 10.1016/S1474-4422(11)70106-5 PMC5511749

[120] Pleger B Tegenthoff M Ragert P. Sensorimotor returning in complex regional pain syndrome parallels pain reduction. Ann Neurol. 2005;57:425–9. 15732114 10.1002/ana.20394

[121] Lebel A Becerra L Wallin D. FMRI reveals distinct CNS processing during symptomatic and recovered complex regional pain syndrome in children. Brain. 2008;131:1854–79. 18567621 10.1093/brain/awn123

[122] Jenkins B Tepper SJ. Neurostimulation for primary headache disorders, part 1: Pathophysiology and anatomy, history of neuromodulation in headache treatment, and review of peripheral neuromodulation in primary headaches. Headache. 2011;51:1254–66. 21815889 10.1111/j.1526-4610.2011.01966.x

[123] May A Bahra A Büchel C. Hypothalamic activation in cluster headache attacks. Lancet. 1998;352:275–8. 9690407 10.1016/S0140-6736(98)02470-2

[124] Sprenger T Boecker H Tolle TR. Specific hypothalamic activation during a spontaneous cluster headache attack. Neurology. 2004;62:516–7. 14872051 10.1212/wnl.62.3.516

[125] Napadow V LaCount L Park K. Intrinsic brain connectivity in fibromyalgia is associated with chronic pain intensity. Arthritis Rheumatol. 2010;6:2545–55. 10.1002/art.27497 PMC2921024 20506181

[126] Jensen KB Loitoile R Kosek E. Patients with fibromyalgia display less functional connectivity in the brain’s pain inhibitory network. Mol Pain. 2012;8:1744–8069. 10.1186/1744-8069-8-32 PMC3404927 22537768

[127] Pedersen LH Scheel-Krüger J Blackburn-Munro G. Amygdala GABA-A receptor involvement in mediating sensory-discriminative and affective-motivational pain responses in a rat model of peripheral nerve injury. Pain. 2007;127:17–26. 16965855 10.1016/j.pain.2006.06.036

[128] Fu Y Han J Ishola T. PKA and ERK, but not PKC, in the amygdala contribute to pain-related synaptic plasticity and behavior. Mol Pain. 2008;4:17441–8069. 10.1186/1744-8069-4-26 PMC2490682 18631385

[129] Campbell JN Meyer RA. Mechanisms of neuropathic pain. Neuron. 2006;52:77–92. 17015228 10.1016/j.neuron.2006.09.021 PMC1810425

[130] Bravo L Llorca-Torralba M Berrocoso E. Monoamines as drug targets in chronic pain: focusing on neuropathic pain. Front Neurosci. 2019;13:155–72. 31942167 10.3389/fnins.2019.01268 PMC6951279

[131] Crofford LJ. Psychological aspects of chronic musculoskeletal pain. Best Pract Res Clin Rheumatol. 2015;29:147–55. 26267008 10.1016/j.berh.2015.04.027 PMC5061342

