# Supplementary material for: Transcranial magnetic stimulation maps the neurophysiology of chronic noncancer pain: A scoping review
Source: Medicine (Baltimore). 2022 Nov 18;101(46):e31774. doi: 10.1097/MD.0000000000031774 (PMC9678597; doi:10.1097/MD.0000000000031774)
Supplement: Supplementary file 1 [file medi-101-e31774-s001.pdf]

Supplementary Table S1. Participant characteristics from included studies.

Study	Chronic Pain Syndrome (ICD-11 Classification)	Age of Patient / Intervention Group*	Age of HCs / Control Group*	Number (patient / intervention group)	Number (HCs / control group)	Number of Females (patients / intervention group)	Number of Females (HCs / control group)	Disease Duration*	Baseline Disease Management	Handedness
Cross-sectional studies										
da Graca-Tarragó et al., 2016a [1]	Knee OA (Chronic Secondary Musculoskeletal Pain)	64.50 ± 7.72 years	34.10 ± 11.64 years	OA: 21	HC: 10	21	—	Inclusion Criteria: > 6 months; 6.73 ± 2.53 years	Psychotropic medications (11); analgesic drugs used > 3 times per week during the past 3 months (14); glucosamine chondroitin (6)	OA: All right-handed; HC: Not specified
Mhalla et al., 2010 [2]	FM (Chronic Primary Pain)	50.8 ± 10.3 years	46.7 ± 11.6 years	FM: 46; Subgroups: those receiving (25) and those not receiving (21) treatment with a psychotropic drug	HC: 21	46	21	Inclusion Criteria: > 6 months; Patients with treatment: 13 ± 12.9 months; Patients without treatment: 14.1 ± 11.9 months	Subgroup receiving treatment, taking ≥ one drug for analgesia, anxiety, and/or depression: tramadol (12), pregabalin (6), amitriptyline (2), duloxetine (2), venlafaxine (4), escitalopram (6), milnacipran (3), clonazepam (10), bromazepam (3), zolpidem (2)	All right-handed
Tang et al., 2019 [3]	CPSP (Chronic Neuropathic Pain)	58.5 ± 8.9 years; Range = 36-74 years	58.9 ± 9.1 years	CPSP: 14	HC: 14	2	2	Inclusion Criteria: > 6 months; 3.3 ± 3.0 years; Range = 0.5-10 years	Antineuralgic agents (10): duloxetine (5), imipramine (4), clonazepam (3), pregabalin (1), and oxcarbamazepine (1).	—

									To avoid the interference of medications on motor cortex excitability and function of somatosensory systems, all CPSP participants who had taken antineuralgic agents were asked to discontinue their medications for ≥ 3 days before the study.	
Turgut & Altun, 2009 [4]	DNP (Chronic Neuropathic Pain)	DNP: 63.9 ± 7.3 years	Pain-free DM: 59.2 ± 11.4 years; HC: 58.3 ± 6.5 years	DNP: 20	Pain-free DM: 50; HC: 30	15	Pain-free DM: 24; HC: 16	Inclusion Criteria: > 6 months; DNP: 63.9 ± 7.3 years; Pain-free DM: 58.2 ± 11.4 years	Insulin: DNP (5), pain-free DM (15)	—
Turton et al., 2007 [5]	CRPS (Chronic Primary Pain)	45 ± 13 years; Range = 16-59 years	45 ± 13 years; Range = 16-59 years	CRPS: 8	HC: 8	7	HC: 7	Range = 0.5-15 years	—	CRPS: Right side affected (5), left side affected (3); HC: Matched to CRPS
Vallence et al., 2013 [6]	CTTH (Chronic Primary Pain)	35 ± 13.2 years	28 ± 8.0 years	CTTH: 11	HC: 18	6	11	Inclusion Criteria: > 1 year	—	CTTH: Not specified; HC: All

										right-handed
van Velzen et al., 2015 [7]	CRPS (Chronic Primary Pain)	51 ± 9.5 years; Range = 34-63 years	SBF: 24 ± 20.5-33.5 years; Range = 19-50 years; HC: 51 ± 9.5 years; Range = 34-63 years	CRPS: 12	SBF: 6; HC: 12	10	SBF: 3; HC: 12	Inclusion Criteria: > 6 months; 88.0 ± 26.9 months; Range = 9-360 months	CRPS patients using centrally acting drugs on day of study (8): tramadol-acetaminophen (1), tramadol (1), etoricoxib (1), pregabalin (3), oxycodone (1), baclofen (1), temazepam (1), amitriptyline (4), gabapentin (1), diazepam (1), ketamine infusion in past month (1)	CRPS: Right-handed (9), left-handed (3); right side affected (4), left side affected (7), both sides affected (1); SBF: Right-handed (5), left-handed (1); right side affected (3), left side affected (3); HC: Right-handed (9), left-handed (3)
Interventional studies										
Bradnam et al., 2016 [8]	SP (Chronic Secondary Musculoskeletal Pain)	Mean = 64.9 years; Range = 49-75 years	Mean = 41.3 years; Range = 20-68 years	SP: 8	HC: 26	7	8	Inclusion Criteria: > 1 year	—	SP: Right side affected (4), left side affected (4)
da Graca-Tarragó et al, 2016b [9]	Knee OA (Chronic Secondary	62.15 ± 7.44 years	66.85 ± 7.53 years	OA, active EIMS: 13	OA, placebo EIMS: 13	13	13	Inclusion Criteria: > 6 months; Active	Psychotropic drugs and analgesics (number not stated;	All right-handed

	Musculoskeletal Pain)							EIMS: 6.67 ± 1.59 years; Placebo EIMS: 6.49 ± 1.48 years	no difference between groups)	
Lefaucheur et al, 2006 [10]	CHP (Chronic Neuropathic Pain)	56.5 ± 2.9 years; Range = 28-75 years	54.8 ± 2.5 years; Range = 33-71 years	22	22	10	12	Range: 2-18 years	Analgesic medication (N): clomipramine (10), fluoxetine (1), morphine sulfate (5), buprenorphine (2), fentanyl (2), acepromazine (2), amitriptyline (3), clonazepam (14), oxazepam (1), lamotrigine (1), tramadol (2), gabapentin (7), carbamazepine (3), dextropropoxyphene (5), codeine (1), paracetamol (5), paroxetine (1), bromazepam (2), clomipramine (1)	All right-handed
Mhalla et al., 2011 [11]	FM (Chronic Primary Pain)	51.8 ± 11.6 years	49.6 ± 10.0 years	FM, active rTMS: n = 20	FM, placebo rTMS: 20	20	20	Inclusion Criteria: > 6 months Active rTMS: 13.0 ± 12.9 years; Placebo rTMS: 14.1 ± 11.9 years	Concomitant medication for pain and sleep disorders were allowed if dose was stable for 1 month before enrollment and throughout study. Medications included analgesics,	All right-handed

									antidepressants, and benzodiazepines (number not stated; no difference between groups)	
Schwenkreis et al., 2003 [12]	PLP (Chronic Neuropathic Pain)	PLP (both memantine and placebo): Median = 62 years; Range = 35-71 years	Median = 35 years; Range = 28-45 years	PLP memantine: 8	PLP placebo: 8; HC: 8	PLP memantine: 1	PLP placebo: 1; HC: Not specified	PLP Memantine: Median (Range) = 17.5 (2-43) years; PLP Placebo: Median (Range) = 24.5 (2-49) years	—	—

*All values are mean ± standard deviation, unless otherwise specified. Abbreviations in order of mention: ICD-11, International Statistical Classification of Diseases and Related Health Problems, 11th Edition [13–15]; TMS, transcranial magnetic stimulation; HC, healthy control; FM, fibromyalgia; CPSP, central post-stroke pain; OA, osteoarthritis; DNP, diabetic neuropathic pain; DM, diabetes mellitus; CRPS, complex regional pain syndrome; CTTH, chronic tension-type headache; SBF, scaphoid bone fracture; SEM, standard error of mean; SP, chronic shoulder pain; EIMS, electrical intramuscular stimulation; CHP, chronic hand pain; rTMS, repetitive transcranial magnetic stimulation; PLP, phantom limb pain.

References for Supplementary Table S1

1. da Graca Tarragó ML, Deitos A, Brietzke AP, et al. Descending Control of Nociceptive Processing in Knee Osteoarthritis Is Associated with Intracortical Disinhibition. *Med (United States)*. 95(17):1-10. doi:10.1097/MD.0000000000003353
2. Mhalla A, de Andrade DC, Baudic S, Perrot S, Bouhassira D. Alteration of cortical excitability in patients with fibromyalgia. *Pain*. 2010;149(3):495-500. doi:10.1016/j.pain.2010.03.009
3. Tang SC, Lee LJH, Jeng JS, et al. Pathophysiology of central poststroke pain motor cortex disinhibition and its clinical and sensory correlates. *Stroke*. 2019;50(10):2851-2857. doi:10.1161/STROKEAHA.119.025692
4. Turgut N, Altun BU. Cortical disinhibition in diabetic patients with neuropathic pain. *Acta Neurol Scand*. 2009;120(6):383-388. doi:10.1111/j.1600-0404.2009.01235.x
5. Turton AJ, McCabe CS, Harris N, Filipovic SR. Sensorimotor integration in Complex Regional Pain Syndrome: A transcranial magnetic stimulation study. *Pain*. 2007;127(3):270-275. doi:10.1016/j.pain.2006.08.021
6. Vallence AM, Smith A, Tabor A, Rolan PE, Ridding MC. Chronic tension-type headache is associated with impaired motor learning. *Cephalalgia*. 2013;33(12):1048-1054. doi:10.1177/0333102413483932
7. Van Velzen GAJ, Marinus J, Van Dijk JG, Van Zwet EW, Schipper IB, Van Hilten JJ. Motor cortical activity during motor tasks is normal in patients with complex regional pain syndrome. *J Pain*. 2015;16(1):87-94. doi:10.1016/j.jpain.2014.10.010
8. Bradnam L, Shanahan EM, Hendy K, et al. Afferent inhibition and cortical silent periods in shoulder primary motor cortex and effect of a suprascapular nerve block in people experiencing chronic shoulder pain. *Clin Neurophysiol*. 2016;127(1):769-778. doi:10.1016/j.clinph.2015.03.012
9. da Graca-Tarragó ML, Deitos A, Brietzke AP, et al. Electrical intramuscular stimulation in osteoarthritis enhances the inhibitory systems in pain processing at cortical and cortical spinal system. *Pain Med (United States)*. 17(5):877-891. doi:10.1111/pme.12930
10. Lefaucheur JP, Drouot X, Ménard-Lefaucheur I, Keravel Y, Nguyen JP. Motor cortex rTMS restores defective intracortical inhibition in chronic neuropathic pain. *Neurology*. 2006;67(9):1568-1574. doi:10.1212/01.wnl.0000242731.10074.3c
11. Mhalla A, Baudic S, De Andrade DC, et al. Long-term maintenance of the analgesic effects of transcranial magnetic stimulation in fibromyalgia. *Pain*. 2011;152(7):1478-1485. doi:10.1016/j.pain.2011.01.034
12. Schwenkreis P, Maier C, Pleger B, et al. NMDA-mediated mechanisms in cortical excitability changes after limb amputation. *Acta Neurol Scand*. 2003;108(3):179-184. doi:10.1034/j.1600-0404.2003.00114.x
13. World Health Organization (WHO). *International Statistical Classification of Diseases and Related Health Problems.* 11th ed.; 2019. https://icd.who.int/.
14. Scholz J, Finnerup NB, Attal N, et al. The IASP classification of chronic pain for ICD-11: Chronic neuropathic pain. *Pain*. 2019;160(1):53-59. doi:10.1097/j.pain.0000000000001365
15. Treede R-D, Rief W, Barke A, et al. A classification of chronic pain for ICD-11. *Pain*. 2015;156(6):1003-1007. doi:10.1097/j.pain.0000000000000160
16. Cleeland CS, Ryan KM. Pain assessment: global use of the Brief Pain Inventory. *Ann Acad Med Singapore*. 1994;23(2):129-138.

17. Burckhardt CS, Clark BD, Bennett RM. The fibromyalgia impact questionnaire: development and validation. *J Rheumatol*. 1991;18:728-733.
18. Beck AT, Ward CH, Mendelson M, Mock J, Erbaugh J. An inventory for measuring depression. *Arch Gen Psychiatry*. 1961;4:561-571.
19. Sullivan MJL, Bishop SR, Pivik J. The Pain Catastrophizing Scale: Development and validation. *Psychol Assess*. 1995;7(4):524–532. doi:10.1037/1040-3590.7.4.524
20. WHOQOL Group. The World Health Organization Quality of Life assessment (WHOQOL): Position paper from the World Health Organization. *Soc Sci Med*. 1995;41:1403-1409.
21. EuroQol Group. *EQ-5D: An Instrument to Describe and Value Health*. http://www.euroqol.org/.
22. Bellamy N, Buchanan WW, Goldsmith CH, Campbell J, Stitt LW. Validation study of WOMAC: a health status instrument for measuring clinically important patient relevant outcomes to antirheumatic drug therapy in patients with osteoarthritis of the hip or knee. *J Rheumatol*. 1988;15(12):1833-1840.
23. Buysse DJ, Reynolds III CF, Monk TH, Berman SR, Kupfer DJ. The Pittsburgh Sleep Quality Index: a new instrument for psychiatric practice and research. *Psychiatry Res*. 1989;28(2):193-213.
24. Bennett M. The LANSS Pain Scale : the Leeds assessment of neuropathic symptoms and signs. 2001;92.
25. Melzack R. The McGill Pain Questionnaire: major properties and scoring methods. *Pain*. 1975;(1):277-299.
26. Harden RN, Bruehl S, Perez RSGM, et al. Development of a severity score for CRPS. *Pain*. 2010;151(3):870-876. doi:10.1016/j.pain.2010.09.031
27. Oerlemans HM, Cup EH, DeBoo T, Goris RJ, Oostendorp RA. The Radboud skills questionnaire: construction and reliability in patients with reflex sympathetic dystrophy of one upper extremity. *Disabil Rehabil*. 2000;22(5):233-245. doi:10.1080/096382800296809
28. Roberts R, Callow N, Hardy L, Markland D, Bringer J. Movement Imagery Ability : Development and Assessment of a Revised Version of the Vividness of Movement Imagery Questionnaire. 2008:200-221.
29. Burke RE, Fahn S, Marsden CD, Bressman SB, Moskowitz C, Friedman J. Validity and reliability of a rating scale for the primary torsion dystonias. *Neurology*. 1985;35(1):73-77. doi:10.1212/wnl.35.1.73
