# Supplementary material for: Transcranial magnetic stimulation maps the neurophysiology of chronic noncancer pain: A scoping review
Source: Medicine (Baltimore). 2022 Nov 18;101(46):e31774. doi: 10.1097/MD.0000000000031774 (PMC9678597; doi:10.1097/MD.0000000000031774)
Supplement: Supplementary file 2 [file medi-101-e31774-s002.pdf]

Supplementary Table S2. Baseline characteristics and design of included studies.

Study	Chronic Pain Syndrome (ICD-11 Classification)	Design	Sample Size	Inclusion Criteria	Exclusion Criteria	Intervention (patients only)	Pain Measures	Additional Clinical Measures
Cross-sectional studies								
da Graca-Tarragó et al., 2016a [1]	Knee OA (Chronic Secondary Musculoskeletal Pain)	CC	21 OA, 10 HC	OA: > 50 years of age; experiencing moderate or intense pain or stiffness in the knee; functional impairments for ≥ 6 months that were not controlled with medical therapy.	OA: Other orthopedic, rheumatologic, neurologic pathology, surgery on affected area in past 6 months, habitual corticosteroid use, BMI > 35, TMS contraindications.<br>HC: Painful condition; use of analgesics or corticosteroids; any rheumatologic, psychiatric, neurologic disorder; alcohol use disorder; use of psychotropic substances during past 6 months; using medications with CNS effects, TMS contradictions.	—	WOMAC;<br>PPT;<br>CPM (cold immersion)	BDI;<br>PSQI;<br>PCS
Mhalla et al., 2010 [2]	FM (Chronic Primary Pain)	CC	46 FM, HC 21	FM: Meet American College criteria for FM with pain > 6 months.<br>Subgroup taking psychotropic drug or on treatment: Taking ≥ one drug for analgesia, anxiety, and/or depression.	FM: Evidence of other inflammatory rheumatic disease, autoimmune disease, other painful disorder, current primary psychiatric condition; history of substance use disorder, seizure, brain injury or surgery, intracranial hypertension, pacemaker, or other metal implant.<br>HC: History of seizure, brain trauma or surgery,	—	BPI	FIQ;<br>BDI;<br>PCS

					intracranial hypertension, abnormal clinical examination, on medication presently or during month preceding testing.			
Tang et al., 2019 [3]	CPSP (Chronic Neuropathic Pain)	CC	14 CPSP, 14 HC	CPSP: Sensory deficits; neuropathic pain occurring after stroke for > 6 months in the extremity contralateral to the stroke hemisphere; preservation of motor function in the extremity contralateral to the stroke hemisphere.	CPSP: Pain from peripheral neuropathy, arthralgia, structural lesion, psychiatric cause; history of seizure, dysphagia, dementia, psychotic, personality disorder; aneurysm clip or pacemaker; surgery following stroke.	—	Pain NRS; Heat pain verbal rating scale	WHOQOL; EQOL5; CHEP; SSEP; QST
Turgut & Altun, 2009 [4]	DNP (Chronic Neuropathic Pain)	CC	20 DNP, 30 Pain-free DM, 50 HC	All DM: Diagnosed using American Diabetes Association criteria.<br>DNP: Neuropathic pain ≥ 6 months, ≥ 12 on LANSS Scale.	All: Alcohol use disorder, exposure to neurotoxic agent, vitamin B12 deficiency, thyroid dysfunction, renal failure, intracranial abnormality, epilepsy, other pain syndrome, severe obesity, edematous limbs, carpal tunnel syndrome,	—	Pain VAS	—

					radiculopathy, plexopathy, mononeuropathy, neuropathic pain, medications with CNS effects.			
Turton et al., 2007 [5]	CRPS (Chronic Primary Pain)	CC	8 CRPS, 8 HC	CRPS: Adults with unilateral upper limb CRPS type I using International Association for the Study of Pain criteria, no impairing contractures, able to tolerate the experimental conditions.<br>HC: No history of CRPS, arthritis, or chronic hand pain.	—	—	Pain VAS	Clinical exam; SSEP
Vallence et al., 2013 [6]	CTTH (Chronic Primary Pain)	CC	11 CTTH, 13 HC	CTTH: Diagnosed using International Classification of Headache Disorders 2nd Ed. > 1 year prior; episodes > 4 hours (if untreated); onset before 50 years of age.	All: TMS contraindications, other neurologic condition, use of prophylactic migraine drugs or any drugs with CNS effects within 8 weeks.<br>CTTH: Medication-overuse headache, concomitant psychiatric disease, other clinically significant pain condition.	—	Pain VAS	Motor learning (ballistic thumb abduction task)

van Velzen et al., 2015 [7]	CRPS (Chronic Primary Pain)	CC	12 CRPS, 6 SBF, 12 HC	CRPS: CRPS of upper limb using Budapest Criteria, loss of voluntary motor control of affected limb for > 6 months; weakness; slowness of movement. SBF: Unilateral SBF immobilized for ≥ 4 weeks.	CRPS: Any relevant neurologic illness, other pain condition, functional impairment of arm. HC: Pain, neurologic disease, other condition impairing hand function.	—	Pain NRS; MPQ; PPT; CSS	RSQ; VMIQ-2; BFMD-RS; Clinical exam
Interventional studies								
Bradnam et al., 2016 [8]	SP (Chronic Secondary Musculoskeletal Pain)	Open label	8 SP, 26 HC	HC: No history of MSK or neurological condition impacting upper limb, neck, or shoulder. SP: Diagnosed with chronic rotator cuff and sub-acromial pathology, subacromial impingement syndrome, or tendinopathy on ultrasound; pain ≥ 1 year.	SP: Rotator cuff tear, unable to perform some degree of lateral rotation of the shoulder.	SSNB with 1 ×40 mg subcutaneous injection of methylprednisolone + 0.5% bupivacaine, after 1% lidocaine injection for local analgesia.	Pain VAS	—
da Graca-Tarragó et al, 2016b [9]	Knee OA (Chronic Secondary Musculoskeletal Pain)	RCT	13 Active, 13 Placebo	Right-handed, aged 50-75 years, moderate to severe pain or stiffness of knee and functional impairment of ≥ 6 months not controlled by medications, reported moderate or severe pain, stiffness,	Other orthopedic, rheumatic, neurologic disease; surgery in affected area in past 6 months, habitual corticosteroid use, uncompensated chronic pathology;	Active vs. placebo EIMS in L1-S3 dermatomes. Acupuncture needles, attached to electroacupuncture device, applied to dermatomes corresponding to L1-S2 nerve roots. Active EIMS involved 1 × 30-	Pain VAS; PPT; CPM (cold immersion)	BDI; PSQI; PCS; WOMAC; Serum BDNF

				and functional impairments (WOMAC); radiographic evidence of knee OA.	acupuncture, BMI > 35, recent knee physiotherapy, unable to read or write.	minute session of 2 Hz electrical stimulation at above acupuncture sites. Placebo EIMS involved 1 × 30-minute session with same electroacupuncture device, acupuncture needles, and sites, but electrical current prevented from passing through needles.		
Lefaucheur et al, 2006 [10]	CHP (Chronic Neuropathic Pain)	RCT	22 CHP, 22 HC	CHP: Chronic, drug-resistant, unilateral neuropathic pain involving at least the hand; HC: No medical history of neurological symptoms or medication use.	History of seizure.	3 × M1 rTMS, separated by ≥ 3 weeks, performed in random order: 1) 20 × 6-second trains of rTMS (54-second intertrain interval) at 10 Hz and 90% RMT using active coil (1,200 pulses); 2) same rTMS protocol using sham stimulation (active coil held at 45° away from skull; 3) 1 × 20-minute train of rTMS at 1 Hz and 90% RMT using active coil (1,200 pulses).	Pain VAS	—
Mhalla et al., 2011 [11]	FM (Chronic Primary Pain)	RCT	20 Active, 20 Placebo	Right-handed, ≥ 18 years of age, FM diagnosed using American College of Rheumatology criteria, pain score ≥ 4 (BPI), persistent pain ≥ 6 months.	Any autoimmune, rheumatic, other painful disorder; primary psychiatric condition; substance use disorder; pregnancy; contraindications to TMS; pain or sleep medications	Active vs. placebo rTMS over left M1. 14 stimulation sessions over 21 weeks, with follow-up at week 25. Induction phase: 1 session/day × 5 consecutive days; Maintenance phase: 1 session/week × 3 weeks, then 3 sessions 1 × every other week (over 6 weeks), then 3 sessions 1	Pain NRS; MPQ; BPI; FIQ	HADS; BDI; PCS

					initiated or not stable over past 1 month.	× per month (over 12 weeks). Active rTMS consisted of 15 series of 10-second pulse trains (50-second intertrain interval) at 10 Hz and 80% RMT (1,500 pulses). Placebo rTMS used coil of identical size, colour, and shape, which emitted a sound like active coil.		
Schwenkreis et al., 2003 [12]	PLP (Chronic Neuropathic Pain)	RCT	8 Active, 8 Placebo	Upper limb amputation, ≥ 12 months of unchanged phantom limb pain.	Any change in pain treatment within previous 4 weeks.	Treatment with memantine capsules daily × 21 days. Dosage of 5 mg on day 1, with 5 mg/day increase up to maximum daily dosage of 30 mg by day 6. Capsules of same colour and size used for placebo and memantine conditions (5 mg⁄capsule).	Pain NRS	—

Abbreviations in order of mention: ICD-11, International Statistical Classification of Diseases and Related Health Problems, 11th Edition [13–15]; FM, fibromyalgia; CC, cross-sectional; HC, healthy control; BPI, Brief Pain Inventory [16]; FIQ, Fibromyalgia Impact Questionnaire [17]; BDI, Beck Depression Inventory [18]; PCS, Pain Catastrophizing Scale [19]; CPSP, central post-stroke pain; NRS, numerical rating scale; WHOQOL, World Health Organization Quality of Life assessment [20]; EQ-5D, European Quality of Life-5 Dimensions [21]; CHEP, contact heat evoked potential; SSEP, somatosensory evoked potential; QST, quantitative sensory testing; OA, osteoarthritis; BMI, body mass index; TMS, transcranial magnetic stimulation; CNS, central nervous system; WOMAC, Western Ontario and McMaster Universities Osteoarthritis Index [22]; PPT, pain pressure threshold; CPM, conditioned pain modulation; PSQI, Pittsburgh Sleep Quality Index [23]; DNP, diabetic neuropathic pain; DM, diabetes mellitus; LANSS, Leeds Assessment of Neuropathic Symptoms and Signs [24]; VAS, visual analog scale; CRPS, complex regional pain syndrome; SBF, scaphoid bone fracture; MPQ, McGill Pain Questionnaire [25]; CSS, CRPS Severity Score [26]; RSQ, Radboud Skills Questionnaire [27];VMIQ-2, Vividness of Movement Imagery Questionnaire-2 [28]; BFMD-RS, Burke-Fahn-Marsden Dystonia Rating Scale [29]; CTTH, chronic tension-type headache; MSK, musculoskeletal; SP, chronic shoulder pain; SSNB, subscapular nerve block; RCT, randomized controlled trial; EIMS, electrical intramuscular stimulation; BDNF, brain-derived neurotrophic factor; CHP, chronic hand pain; M1, primary motor cortex; rTMS, repetitive transcranial magnetic stimulation; RMT, resting motor threshold; PLP, phantom limb pain.

References for Supplementary Table 2

1. da Graca Tarragó ML, Deitos A, Brietzke AP, et al. Descending Control of Nociceptive Processing in Knee Osteoarthritis Is Associated with Intracortical Disinhibition. *Med (United States)*. 95(17):1-10. doi:10.1097/MD.0000000000003353
2. Mhalla A, de Andrade DC, Baudic S, Perrot S, Bouhassira D. Alteration of cortical excitability in patients with fibromyalgia. *Pain*. 2010;149(3):495-500. doi:10.1016/j.pain.2010.03.009
3. Tang SC, Lee LJH, Jeng JS, et al. Pathophysiology of central poststroke pain motor cortex disinhibition and its clinical and sensory correlates. *Stroke*. 2019;50(10):2851-2857. doi:10.1161/STROKEAHA.119.025692
4. Turgut N, Altun BU. Cortical disinhibition in diabetic patients with neuropathic pain. *Acta Neurol Scand*. 2009;120(6):383-388. doi:10.1111/j.1600-0404.2009.01235.x
5. Turton AJ, McCabe CS, Harris N, Filipovic SR. Sensorimotor integration in Complex Regional Pain Syndrome: A transcranial magnetic stimulation study. *Pain*. 2007;127(3):270-275. doi:10.1016/j.pain.2006.08.021
6. Vallence AM, Smith A, Tabor A, Rolan PE, Ridding MC. Chronic tension-type headache is associated with impaired motor learning. *Cephalalgia*. 2013;33(12):1048-1054. doi:10.1177/0333102413483932
7. Van Velzen GAJ, Marinus J, Van Dijk JG, Van Zwet EW, Schipper IB, Van Hilten JJ. Motor cortical activity during motor tasks is normal in patients with complex regional pain syndrome. *J Pain*. 2015;16(1):87-94. doi:10.1016/j.jpain.2014.10.010
8. Bradnam L, Shanahan EM, Hendy K, et al. Afferent inhibition and cortical silent periods in shoulder primary motor cortex and effect of a suprascapular nerve block in people experiencing chronic shoulder pain. *Clin Neurophysiol*. 2016;127(1):769-778. doi:10.1016/j.clinph.2015.03.012
9. da Graca-Tarragó ML, Deitos A, Brietzke AP, et al. Electrical intramuscular stimulation in osteoarthritis enhances the inhibitory systems in pain processing at cortical and cortical spinal system. *Pain Med (United States)*. 17(5):877-891. doi:10.1111/pme.12930
10. Lefaucheur JP, Drouot X, Ménard-Lefaucheur I, Keravel Y, Nguyen JP. Motor cortex rTMS restores defective intracortical inhibition in chronic neuropathic pain. *Neurology*. 2006;67(9):1568-1574. doi:10.1212/01.wnl.0000242731.10074.3c
11. Mhalla A, Baudic S, De Andrade DC, et al. Long-term maintenance of the analgesic effects of transcranial magnetic stimulation in fibromyalgia. *Pain*. 2011;152(7):1478-1485. doi:10.1016/j.pain.2011.01.034
12. Schwenkreis P, Maier C, Pleger B, et al. NMDA-mediated mechanisms in cortical excitability changes after limb amputation. *Acta Neurol Scand*. 2003;108(3):179-184. doi:10.1034/j.1600-0404.2003.00114.x
13. World Health Organization (WHO). *International Statistical Classification of Diseases and Related Health Problems.* 11th ed.; 2019. https://icd.who.int/.
14. Scholz J, Finnerup NB, Attal N, et al. The IASP classification of chronic pain for ICD-11: Chronic neuropathic pain. *Pain*. 2019;160(1):53-59. doi:10.1097/j.pain.0000000000001365
15. Treede R-D, Rief W, Barke A, et al. A classification of chronic pain for ICD-11. *Pain*. 2015;156(6):1003-1007. doi:10.1097/j.pain.0000000000000160
16. Cleeland CS, Ryan KM. Pain assessment: global use of the Brief Pain Inventory. *Ann Acad Med Singapore*. 1994;23(2):129-138.

17. Burckhardt CS, Clark BD, Bennett RM. The fibromyalgia impact questionnaire: development and validation. *J Rheumatol*. 1991;18:728-733.
18. Beck AT, Ward CH, Mendelson M, Mock J, Erbaugh J. An inventory for measuring depression. *Arch Gen Psychiatry*. 1961;4:561-571.
19. Sullivan MJL, Bishop SR, Pivik J. The Pain Catastrophizing Scale: Development and validation. *Psychol Assess*. 1995;7(4):524–532. doi:10.1037/1040-3590.7.4.524
20. WHOQOL Group. The World Health Organization Quality of Life assessment (WHOQOL): Position paper from the World Health Organization. *Soc Sci Med*. 1995;41:1403-1409.
21. EuroQol Group. *EQ-5D: An Instrument to Describe and Value Health*. http://www.euroqol.org/.
22. Bellamy N, Buchanan WW, Goldsmith CH, Campbell J, Stitt LW. Validation study of WOMAC: a health status instrument for measuring clinically important patient relevant outcomes to antirheumatic drug therapy in patients with osteoarthritis of the hip or knee. *J Rheumatol*. 1988;15(12):1833-1840.
23. Buysse DJ, Reynolds III CF, Monk TH, Berman SR, Kupfer DJ. The Pittsburgh Sleep Quality Index: a new instrument for psychiatric practice and research. *Psychiatry Res*. 1989;28(2):193-213.
24. Bennett M. The LANSS Pain Scale : the Leeds assessment of neuropathic symptoms and signs. 2001;92.
25. Melzack R. The McGill Pain Questionnaire: major properties and scoring methods. *Pain*. 1975;(1):277-299.
26. Harden RN, Bruehl S, Perez RSGM, et al. Development of a severity score for CRPS. *Pain*. 2010;151(3):870-876. doi:10.1016/j.pain.2010.09.031
27. Oerlemans HM, Cup EH, DeBoo T, Goris RJ, Oostendorp RA. The Radboud skills questionnaire: construction and reliability in patients with reflex sympathetic dystrophy of one upper extremity. *Disabil Rehabil*. 2000;22(5):233-245. doi:10.1080/096382800296809
28. Roberts R, Callow N, Hardy L, Markland D, Bringer J. Movement Imagery Ability : Development and Assessment of a Revised Version of the Vividness of Movement Imagery Questionnaire. 2008:200-221.
29. Burke RE, Fahn S, Marsden CD, Bressman SB, Moskowitz C, Friedman J. Validity and reliability of a rating scale for the primary torsion dystonias. *Neurology*. 1985;35(1):73-77. doi:10.1212/wnl.35.1.73
