# Supplementary material for: Transcranial magnetic stimulation maps the neurophysiology of chronic noncancer pain: A scoping review
Source: Medicine (Baltimore). 2022 Nov 18;101(46):e31774. doi: 10.1097/MD.0000000000031774 (PMC9678597; doi:10.1097/MD.0000000000031774)
Supplement: Supplementary file 3 [file medi-101-e31774-s003.pdf]

Supplementary Table S3. TMS protocols and methods used in included studies.

Study	Target Muscle	Target Stability	EMG Electrodes	Coil Type	TMS Outcomes	Stimulator Intensity	Muscle Relaxation / Contraction	Method to Normalize TMS Outcomes	Determination of Hotspot	IPI	CSI (paired pulse)	TSI (paired pulse)	ISI (paired pulse)
Cross-sectional studies													
da Graca-Tarragó et al., 2016a [1]	Right FDI	Marked on scalp	Belly-tendon montage	F8	RMT; MEP; SICI; ICF; CSP	130% RMT	CSP: Active, 10% MVC, monitoring not specified	RMT: % MSO; MEP: Raw peak-to-peak amplitude; SICI and ICF: Conditioned MEP normalized to unconditioned MEP; CSP: Duration	—	8 s	80% RMT	130% RMT, adjusted to elicit1 mV MEP	SICI: 2 ms; ICF: 12 ms
Mhalla et al., 2010 [2]	FDI (not specified)	—	—	F8	RMT; MEP; SICI; ICF	120% and 140% RMT	Relaxed: monitoring not specified	RMT: % MSO; MEP: Ratio of 140% RMT/120 % RMT MEP amplitude; SICI and ICF: Conditioned MEP normalized to unconditioned MEP	—	—	80% RMT	120% RMT	SICI: 2 and 4 ms; ICF: 10 and 15 ms

Tang et al., 2019 [3]	Bilateral FDI	Marked on scalp	Belly-tendon montage	F8	RMT; MEP; SICI; ICF; SAI; LAI	125% RMT	Relaxed: acoustic EMG feedback	RMT: % MSO; MEP: Raw peak-to-peak amplitude; SICI and ICF: Conditioned MEP normalized to unconditioned MEP; SAI and LAI: Conditioned MEP normalized to unconditioned MEP	Site where stimulation elicited largest MEPs in target muscle	—	SICI and ICF: 70% RMT; SAI and LAI: Peripheral nerve stimulation at 3 × perceptual threshold	125% RMT	SICI: 3, 5, 7 ms; ICF 10, 15, 20 ms; SAI 10, 20, 30, 40 ms; LAI: 100, 200, 300, 400 ms
Turgut & Altun, 2009 [4]	Left FDI	Marked on scalp	—	C	RMT; MEP; CSP	140% RMT	RMT: Relaxed, monitoring not specified; CSP: Active, MVC, monitoring not specified	RMT: % MSO; MEP: Raw peak-to-peak amplitude; CSP: Duration	Site where stimulation elicited largest MEPs in target muscle	—	—	—	—
Turton et al., 2007 [5]	APB of affected upper extremity (CRPS) or matching side (HC)	Marked on scalp	Belly-tendon montage	F8	RMT; MEP; SAI	120% RMT	Relaxed: monitoring not specified	RMT: % MSO MEP: Raw peak-to-peak amplitude; SAI: Conditione	Site where stimulation elicited largest MEPs in target muscle	5 s	Peripheral nerve stimulation to evoke a visible thumb twitch	120% RMT	SSEP N20 latency

								d MEP normalized to unconditioned MEP					
Vallence et al., 2013 [6]	Left FDI	Marked on scalp	Belly-tendon montage	F8	RMT; MEP	1 mV MEP intensity	Relaxed: monitoring not specified	RMT: % MSO; MEP: Raw peak-to-peak amplitude	Site where stimulation elicited consistent MEPs in target muscle	7 s	—	—	—
van Velzen et al., 2015 [7]	Bilateral FDI	Neuronavigation	—	F8	RMT; MEP-RC	80%, 90%, 100%, 110%, 120%, and 130% 1 mV MEP intensity	Relaxed, Motor Observation / Motor Imagery: monitoring not specified	RMT: % MSO; MEP: Raw peak-to-peak amplitude	Site where lowest stimulus intensity was needed to evoke a 100 µV MEP in target muscle	4-6 s	—	—	—
Interventional studies													
Bradnam et al., 2016 [8]	Injured infraspinatus muscle (SP) or dominant infraspinatus muscle (HC)	Marked on scalp	Electrodes 3 cm below midpoint of spine of scapula, 1 cm apart, and aligned with direction infraspinatus fibres; reference	F8	AMT; MEP; MEP_{max}; 50%MEPmax; CSP; SAI	120% AMT	Active: contraction not specified, monitoring not specified	AMT: % MSO; MEP: Area normalized to pre-stimulus EMG; CSP: Duration; SAI: Conditioned MEP normalized to	Site where stimulation elicited largest MEPs in target muscle	—	Peripheral nerve stimulation at 80% motor threshold	120% AMT	20, 30, 40 ms

			electrode over acromion process					unconditioned MEP				
da Graca-Tarragó et al, 2016b [9]	Left FDI	Marked on scalp	Belly-tendon montage	F8	RMT; MEP; SICI; ICF; CSP	130% RMT	RMT/SICI/ICF: Resting, monitoring not specified; MEP/CSP: Active, 10% MVC, monitoring not specified	RMT: % MSO; MEP: Raw peak-to-peak amplitude; SICI and ICF: Conditioned MEP normalized to unconditioned MEP; CSP: Duration	Site where lowest motor threshold intensity was required to elicit an acceptable response in at least 50% trials	8 s	80% RMT	130% RMT	SICI: 2 ms; ICF: 12 ms
Lefaucheur et al, 2006 [10]	FDI (side not specified)	Marked on scalp	Belly-tendon montage	F8	RMT; MEP; SICI; ICF; CSP	—	RMT/ICI/ICF: Relaxed, monitoring not specified MEP/CSP: Active, MVC, monitoring not specified	RMT: % MSO; MEP: Ratio of 140% RMT/120 % RMT MEP amplitude; SICI and ICF: Conditioned MEP normalized to	—	—	80% RMT	120% RMT	ICI: 2 and 4 ms: ICF: 10 and 15 ms

								unconditioned MEP; CSP: Duration					
Mhalla et al., 2011 [11]	Dominant FDI	—	—	F8	RMT; MEP; SICI; ICF	120% and 140% RMT	Relaxed: monitoring not specified	RMT: %MSO; MEP: Ratio of 140% RMT/120% RMT MEP amplitude; SICI and ICF: Conditioned MEP normalized to unconditioned MEP	—	—	80% RMT	120% RMT	SICI: 2 and 4 ms; ICF: 10 and 15 ms
Schwenkreis et al., 2003 [12]	Muscle proximal to amputation stump (deltoid for upper arm amputation, biceps brachii for forearm amputation, FDI for finger	Coil centred over vertex	—	C	RMT; MEP; SICI; ICF	—	Relaxed: acoustic EMG feedback	RMT: %MSO MEP: Raw peak-to-peak amplitude; SICI and ICF: Conditioned MEP normalized to unconditioned MEP	—	—	80% RMT	Adjusted to elicit 0.5-1 mV MEP	ICI: 1, 2, 3, 4, 5 ms ICF: 6, 8, 10, 15, 20 ms

	amputation)	

Abbreviations in order of mention: TMS, transcranial magnetic stimulation; EMG, electromyogram; IPI, inter-pulse interval; CSI, conditioning stimulus intensity; TSI, test stimulus intensity; ISI, interstimulus interval; FDI, first dorsal interosseus muscle; F8, figure-of-eight; RMT, resting motor threshold; MEP, motor evoked potential; SICI, short-interval intracortical inhibition; ICF, intracortical facilitation; MSO, maximal stimulator output; SAI, short-latency afferent inhibition; LAI, long-latency afferent inhibition; CSP, cortical silent period; MVC, maximum voluntary isometric contraction; C, circular; CRPS, complex regional pain syndrome; HC, healthy control; SSEP, somatosensory evoked potential; N20, negative deflection in EEG trace over primary somatosensory cortex at ~20 ms post-stimulation (first cortical response to afferent somatosensory volley); APB, abductor pollicis brevis muscle; SP, chronic shoulder pain; AMT, active motor threshold.

References for Supplementary Table 3

1. da Graca Tarragó ML, Deitos A, Brietzke AP, et al. Descending Control of Nociceptive Processing in Knee Osteoarthritis Is Associated with Intracortical Disinhibition. *Med (United States)*. 95(17):1-10. doi:10.1097/MD.0000000000003353
2. Mhalla A, de Andrade DC, Baudic S, Perrot S, Bouhassira D. Alteration of cortical excitability in patients with fibromyalgia. *Pain*. 2010;149(3):495-500. doi:10.1016/j.pain.2010.03.009
3. Tang SC, Lee LJH, Jeng JS, et al. Pathophysiology of central poststroke pain motor cortex disinhibition and its clinical and sensory correlates. *Stroke*. 2019;50(10):2851-2857. doi:10.1161/STROKEAHA.119.025692
4. Turgut N, Altun BU. Cortical disinhibition in diabetic patients with neuropathic pain. *Acta Neurol Scand*. 2009;120(6):383-388. doi:10.1111/j.1600-0404.2009.01235.x
5. Turton AJ, McCabe CS, Harris N, Filipovic SR. Sensorimotor integration in Complex Regional Pain Syndrome: A transcranial magnetic stimulation study. *Pain*. 2007;127(3):270-275. doi:10.1016/j.pain.2006.08.021
6. Vallence AM, Smith A, Tabor A, Rolan PE, Ridding MC. Chronic tension-type headache is associated with impaired motor learning. *Cephalalgia*. 2013;33(12):1048-1054. doi:10.1177/0333102413483932
7. Van Velzen GAJ, Marinus J, Van Dijk JG, Van Zwet EW, Schipper IB, Van Hilten JJ. Motor cortical activity during motor tasks is normal in patients with complex regional pain syndrome. *J Pain*. 2015;16(1):87-94. doi:10.1016/j.jpain.2014.10.010
8. Bradnam L, Shanahan EM, Hendy K, et al. Afferent inhibition and cortical silent periods in shoulder primary motor cortex and effect of a suprascapular nerve block in people experiencing chronic shoulder pain. *Clin Neurophysiol*. 2016;127(1):769-778. doi:10.1016/j.clinph.2015.03.012
9. da Graca-Tarragó ML, Deitos A, Brietzke AP, et al. Electrical intramuscular stimulation in osteoarthritis enhances the inhibitory systems in pain processing at cortical and cortical spinal system. *Pain Med (United States)*. 17(5):877-891. doi:10.1111/pme.12930
10. Lefaucheur JP, Drouot X, Ménard-Lefaucheur I, Keravel Y, Nguyen JP. Motor cortex rTMS restores defective intracortical inhibition in chronic neuropathic pain. *Neurology*. 2006;67(9):1568-1574. doi:10.1212/01.wnl.0000242731.10074.3c
11. Mhalla A, Baudic S, De Andrade DC, et al. Long-term maintenance of the analgesic effects of transcranial magnetic stimulation in fibromyalgia. *Pain*. 2011;152(7):1478-1485. doi:10.1016/j.pain.2011.01.034
12. Schwenkreis P, Maier C, Pleger B, et al. NMDA-mediated mechanisms in cortical excitability changes after limb amputation. *Acta Neurol Scand*. 2003;108(3):179-184. doi:10.1034/j.1600-0404.2003.00114.x
13. World Health Organization (WHO). *International Statistical Classification of Diseases and Related Health Problems.* 11th ed.; 2019. https://icd.who.int/.
14. Scholz J, Finnerup NB, Attal N, et al. The IASP classification of chronic pain for ICD-11: Chronic neuropathic pain. *Pain*. 2019;160(1):53-59. doi:10.1097/j.pain.0000000000001365
15. Treede R-D, Rief W, Barke A, et al. A classification of chronic pain for ICD-11. *Pain*. 2015;156(6):1003-1007. doi:10.1097/j.pain.0000000000000160
16. Cleeland CS, Ryan KM. Pain assessment: global use of the Brief Pain Inventory. *Ann Acad Med Singapore*. 1994;23(2):129-138.

17. Burckhardt CS, Clark BD, Bennett RM. The fibromyalgia impact questionnaire: development and validation. *J Rheumatol*. 1991;18:728-733.
18. Beck AT, Ward CH, Mendelson M, Mock J, Erbaugh J. An inventory for measuring depression. *Arch Gen Psychiatry*. 1961;4:561-571.
19. Sullivan MJL, Bishop SR, Pivik J. The Pain Catastrophizing Scale: Development and validation. *Psychol Assess*. 1995;7(4):524–532. doi:10.1037/1040-3590.7.4.524
20. WHOQOL Group. The World Health Organization Quality of Life assessment (WHOQOL): Position paper from the World Health Organization. *Soc Sci Med*. 1995;41:1403-1409.
21. EuroQol Group. *EQ-5D: An Instrument to Describe and Value Health*. http://www.euroqol.org/.
22. Bellamy N, Buchanan WW, Goldsmith CH, Campbell J, Stitt LW. Validation study of WOMAC: a health status instrument for measuring clinically important patient relevant outcomes to antirheumatic drug therapy in patients with osteoarthritis of the hip or knee. *J Rheumatol*. 1988;15(12):1833-1840.
23. Buysse DJ, Reynolds III CF, Monk TH, Berman SR, Kupfer DJ. The Pittsburgh Sleep Quality Index: a new instrument for psychiatric practice and research. *Psychiatry Res*. 1989;28(2):193-213.
24. Bennett M. The LANSS Pain Scale : the Leeds assessment of neuropathic symptoms and signs. 2001;92.
25. Melzack R. The McGill Pain Questionnaire: major properties and scoring methods. *Pain*. 1975;(1):277-299.
26. Harden RN, Bruehl S, Perez RSGM, et al. Development of a severity score for CRPS. *Pain*. 2010;151(3):870-876. doi:10.1016/j.pain.2010.09.031
27. Oerlemans HM, Cup EH, DeBoo T, Goris RJ, Oostendorp RA. The Radboud skills questionnaire: construction and reliability in patients with reflex sympathetic dystrophy of one upper extremity. *Disabil Rehabil*. 2000;22(5):233-245. doi:10.1080/096382800296809
28. Roberts R, Callow N, Hardy L, Markland D, Bringer J. Movement Imagery Ability : Development and Assessment of a Revised Version of the Vividness of Movement Imagery Questionnaire. 2008:200-221.
29. Burke RE, Fahn S, Marsden CD, Bressman SB, Moskowitz C, Friedman J. Validity and reliability of a rating scale for the primary torsion dystonias. *Neurology*. 1985;35(1):73-77. doi:10.1212/wnl.35.1.73
