# Supplementary material for: Transcranial magnetic stimulation maps the neurophysiology of chronic noncancer pain: A scoping review
Source: Medicine (Baltimore). 2022 Nov 18;101(46):e31774. doi: 10.1097/MD.0000000000031774 (PMC9678597; doi:10.1097/MD.0000000000031774)
Supplement: Supplementary file 4 [file medi-101-e31774-s004.pdf]

Supplementary Table S4. TMS results from included studies and correlations with clinical outcomes.

Study	Chronic Pain Syndrome (ICD-11 Classification)	AMT	RMT	MEP	SICI	ICF	CSP	SAI	LAI	Correlations
Cross-sectional studies										
da Graca-Tarragó et al., 2016a [1]	Knee OA (Chronic Secondary Musculoskeletal Pain)	—	↑ OA	NS	NS	NS	NS	—	—	↑ CSP and ↓ pain NRS (*r* = –0.72)
Mhalla et al., 2010 [2]	FM (Chronic Primary Pain)	—	↑ FM	↓ FM	↓ FM	↓ FM	—	—	—	↓ right hemisphere ICF and ↓ FIQ (*r* = +0.41); ↓ left hemisphere SICI and ↓ PCS (*r* = +0.42); ↓ left hemisphere SICI and ↓ BDI (*r* = +0.32)
Tang et al., 2019 [3]	CPSP (Chronic Neuropathic Pain)	—	NS	NS	↓ in stroke vs. unaffected hemisphere of CPSP, but not HC	↑ in stroke vs. unaffected hemisphere of CPSP, but not HC	—	NS	NS	↑ SICI and ↓ EQ-5D (*r* not specified); ↑ SICI and ↑ symptomatic to asymptomatic ratio of CHEP N20 latency (R^2 not specified); ↑ SICI and ↑ symptomatic to asymptomatic ratios of SSEP N20 latency (R^2 not specified)

Turgut & Altun, 2009 [4]	DNP (Chronic Neuropathic Pain)	—	↓ DNP	NS	—	—	↓ DNP	—	—	NS
Turton et al., 2007 [5]	CRPS (Chronic Primary Pain)	—	NS	NS	—	—	—	NS	—	—
Vallence et al., 2013 [6]	CTTH (Chronic Primary Pain)	—	NS	↑ 10 and 20 minutes post-motor training vs. baseline in HC but not CTTH	—	—	—	—	—	NS
van Velzen et al., 2015 [7]	CRPS (Chronic Primary Pain)	—	NS	↑ after motor imagery vs. motor observation in CRPS and HC but not SBF	—	—	—	—	—	↑ MEP amplitude during motor imagery and ↓ VMIQ-2 (r = –0.26)
Interventional studies										
Bradnam et al., 2016 [8]	SP (Chronic Secondary Musculoskeletal Pain)	↑ SP	—	NS	—	—	↑ SP<br>↓ 30 minutes after SSNB;<br>↑ 1 week after SSNB	↑ 30 minutes after SSNB	—	↑ AMT and ↑ pain VAS at baseline (R^2 = 0.71); ↓ SAI and ↓ pain VAS from baseline to 30 minutes post-SSNB (R^2 = 0.57)
da Graca-Tarragó et al, 2016b [9]	Knee OA (Chronic Secondary Musculoskeletal Pain)	—	NS	↓ after EIMS	NS	↓ after EIMS	↑ after EIMS	—	—	NS

Lefaucheur et al, 2006 [10]	CHP (Chronic Neuropathic Pain)	—	NS	NS	↓ in painful vs. painless hand in CHP; ↑ after rTMS	NS	↓ CHP	—	—	↓ CSP duration of painful hand and ↑ baseline pain VAS (*r* = –0.33); ↑ SICI and ↓ pain VAS after rTMS (*r* = –0.56)
Mhalla et al., 2011 [11]	FM (Chronic Primary Pain)	—	NS	NS	↑ after rTMS	↑ after rTMS	—	—	—	↑ SICI and ↑ PCS at baseline (*r* = +0.37); ↓ SICI and ↓ pain NRS after rTMS (*r* = +0.47 to +0.71) in active but not placebo; ↓ ICF and ↓ FIQ after rTMS (*r* = +0.52) in active but not placebo; ↓ ICF and ↓ PCS after rTMS (*r* = +0.51) in active but not placebo
Schwenkreis et al., 2003 [12]	PLP (Chronic Neuropathic Pain)	—	NS	NS	↓ PLP; ↓ after memantine	↓ after memantine	—	—	—	NS

Abbreviations in order of mention: TMS, transcranial magnetic stimulation; ICD-11, International Statistical Classification of Diseases and Related Health Problems, 11th Edition [13–15]; AMT, active motor threshold; RMT, resting motor threshold; MEP, motor evoked potential; SICI, short-interval intracortical inhibition; ICF, intracortical facilitation; CSP, cortical silent period; SAI, short-latency afferent inhibition; LAI, long-latency afferent inhibition; FM, fibromyalgia; FIQ, Fibromyalgia Impact Questionnaire [17]; PCS, Pain Catastrophizing Scale [19]; BDI, Beck Depression Inventory [18]; CPSP, central post-stroke pain; NS, not statistically significant at $p < .05$; HC, healthy control; EQ-5D, European Quality of Life-5 Dimensions [21]; CHEP, contact heat evoked potential; N20, negative deflection in EEG trace over primary somatosensory cortex at ~20 ms post-stimulation (first cortical response to afferent somatosensory volley); SSEP, somatosensory evoked potential; OA, osteoarthritis; NRS, numerical rating scale; DNP, diabetic neuropathic pain; CRPS, complex regional pain syndrome; CTTH, chronic tension-type headache; SBF, scaphoid bone fracture; Vividness of Movement Imagery Questionnaire-2 [28]; SP, chronic shoulder pain; SSNB, subscapular nerve block; VAS, visual analog scale; EIMS, electrical intramuscular stimulation; CHP, chronic hand pain; rTMS, repetitive transcranial magnetic stimulation; PLP, phantom limb pain.

References for Supplementary Table 4

1. da Graca Tarragó ML, Deitos A, Brietzke AP, et al. Descending Control of Nociceptive Processing in Knee Osteoarthritis Is Associated with Intracortical Disinhibition. *Med (United States)*. 95(17):1-10. doi:10.1097/MD.0000000000003353
2. Mhalla A, de Andrade DC, Baudic S, Perrot S, Bouhassira D. Alteration of cortical excitability in patients with fibromyalgia. *Pain*. 2010;149(3):495-500. doi:10.1016/j.pain.2010.03.009
3. Tang SC, Lee LJH, Jeng JS, et al. Pathophysiology of central poststroke pain motor cortex disinhibition and its clinical and sensory correlates. *Stroke*. 2019;50(10):2851-2857. doi:10.1161/STROKEAHA.119.025692
4. Turgut N, Altun BU. Cortical disinhibition in diabetic patients with neuropathic pain. *Acta Neurol Scand*. 2009;120(6):383-388. doi:10.1111/j.1600-0404.2009.01235.x
5. Turton AJ, McCabe CS, Harris N, Filipovic SR. Sensorimotor integration in Complex Regional Pain Syndrome: A transcranial magnetic stimulation study. *Pain*. 2007;127(3):270-275. doi:10.1016/j.pain.2006.08.021
6. Vallence AM, Smith A, Tabor A, Rolan PE, Ridding MC. Chronic tension-type headache is associated with impaired motor learning. *Cephalalgia*. 2013;33(12):1048-1054. doi:10.1177/0333102413483932
7. Van Velzen GAJ, Marinus J, Van Dijk JG, Van Zwet EW, Schipper IB, Van Hilten JJ. Motor cortical activity during motor tasks is normal in patients with complex regional pain syndrome. *J Pain*. 2015;16(1):87-94. doi:10.1016/j.jpain.2014.10.010
8. Bradnam L, Shanahan EM, Hendy K, et al. Afferent inhibition and cortical silent periods in shoulder primary motor cortex and effect of a suprascapular nerve block in people experiencing chronic shoulder pain. *Clin Neurophysiol*. 2016;127(1):769-778. doi:10.1016/j.clinph.2015.03.012
9. da Graca-Tarragó ML, Deitos A, Brietzke AP, et al. Electrical intramuscular stimulation in osteoarthritis enhances the inhibitory systems in pain processing at cortical and cortical spinal system. *Pain Med (United States)*. 17(5):877-891. doi:10.1111/pme.12930
10. Lefaucheur JP, Drouot X, Ménard-Lefaucheur I, Keravel Y, Nguyen JP. Motor cortex rTMS restores defective intracortical inhibition in chronic neuropathic pain. *Neurology*. 2006;67(9):1568-1574. doi:10.1212/01.wnl.0000242731.10074.3c
11. Mhalla A, Baudic S, De Andrade DC, et al. Long-term maintenance of the analgesic effects of transcranial magnetic stimulation in fibromyalgia. *Pain*. 2011;152(7):1478-1485. doi:10.1016/j.pain.2011.01.034
12. Schwenkreis P, Maier C, Pleger B, et al. NMDA-mediated mechanisms in cortical excitability changes after limb amputation. *Acta Neurol Scand*. 2003;108(3):179-184. doi:10.1034/j.1600-0404.2003.00114.x
13. World Health Organization (WHO). *International Statistical Classification of Diseases and Related Health Problems.* 11th ed.; 2019. https://icd.who.int/.
14. Scholz J, Finnerup NB, Attal N, et al. The IASP classification of chronic pain for ICD-11: Chronic neuropathic pain. *Pain*. 2019;160(1):53-59. doi:10.1097/j.pain.0000000000001365
15. Treede R-D, Rief W, Barke A, et al. A classification of chronic pain for ICD-11. *Pain*. 2015;156(6):1003-1007. doi:10.1097/j.pain.0000000000000160
16. Cleeland CS, Ryan KM. Pain assessment: global use of the Brief Pain Inventory. *Ann Acad Med Singapore*. 1994;23(2):129-138.

17. Burckhardt CS, Clark BD, Bennett RM. The fibromyalgia impact questionnaire: development and validation. *J Rheumatol*. 1991;18:728-733.
18. Beck AT, Ward CH, Mendelson M, Mock J, Erbaugh J. An inventory for measuring depression. *Arch Gen Psychiatry*. 1961;4:561-571.
19. Sullivan MJL, Bishop SR, Pivik J. The Pain Catastrophizing Scale: Development and validation. *Psychol Assess*. 1995;7(4):524–532. doi:10.1037/1040-3590.7.4.524
20. WHOQOL Group. The World Health Organization Quality of Life assessment (WHOQOL): Position paper from the World Health Organization. *Soc Sci Med*. 1995;41:1403-1409.
21. EuroQol Group. *EQ-5D: An Instrument to Describe and Value Health*. http://www.euroqol.org/.
22. Bellamy N, Buchanan WW, Goldsmith CH, Campbell J, Stitt LW. Validation study of WOMAC: a health status instrument for measuring clinically important patient relevant outcomes to antirheumatic drug therapy in patients with osteoarthritis of the hip or knee. *J Rheumatol*. 1988;15(12):1833-1840.
23. Buysse DJ, Reynolds III CF, Monk TH, Berman SR, Kupfer DJ. The Pittsburgh Sleep Quality Index: a new instrument for psychiatric practice and research. *Psychiatry Res*. 1989;28(2):193-213.
24. Bennett M. The LANSS Pain Scale : the Leeds assessment of neuropathic symptoms and signs. 2001;92.
25. Melzack R. The McGill Pain Questionnaire: major properties and scoring methods. *Pain*. 1975;(1):277-299.
26. Harden RN, Bruehl S, Perez RSGM, et al. Development of a severity score for CRPS. *Pain*. 2010;151(3):870-876. doi:10.1016/j.pain.2010.09.031
27. Oerlemans HM, Cup EH, DeBoo T, Goris RJ, Oostendorp RA. The Radboud skills questionnaire: construction and reliability in patients with reflex sympathetic dystrophy of one upper extremity. *Disabil Rehabil*. 2000;22(5):233-245. doi:10.1080/096382800296809
28. Roberts R, Callow N, Hardy L, Markland D, Bringer J. Movement Imagery Ability : Development and Assessment of a Revised Version of the Vividness of Movement Imagery Questionnaire. 2008:200-221.
29. Burke RE, Fahn S, Marsden CD, Bressman SB, Moskowitz C, Friedman J. Validity and reliability of a rating scale for the primary torsion dystonias. *Neurology*. 1985;35(1):73-77. doi:10.1212/wnl.35.1.73
